# Heterogeneity in Meat Food Groups Can Meaningfully Alter Population-Level Intake Estimates of Red Meat and Poultry

**DOI:** 10.3389/fnut.2021.778369

**Published:** 2021-12-15

**Authors:** Lauren E. O'Connor, Kirsten A. Herrick, Ruth Parsons, Jill Reedy

**Affiliations:** ^1^Risk Factor Assessment Branch, Epidemiology and Genomics Research Program, Division of Cancer Control and Population Sciences, National Cancer Institute, National Institutes of Health, Rockville, MD, United States; ^2^Information Management Services, Inc., Rockville, MD, United States

**Keywords:** dietary assessment, standardization, food groups, nutrition surveillance, nutrition epidemiology, U.S. populations

## Abstract

Heterogeneity in meat food groups hinders interpretation of research regarding meat intake and chronic disease risk. Our objective was to investigate how heterogeneity in red meat (RM) and poultry food groups influences US population intake estimates. Based on a prior systematic review, we created an ontology of methods used to estimate RM [1= unprocessed RM; 2 (reference)= unprocessed RM + processed RM; 3= unprocessed RM + processed RM + processed poultry; and 4=unprocessed RM + processed RM + processed poultry + chicken patties/nuggets/tenders (PNT)] and three for poultry [A=unprocessed poultry; B= unprocessed poultry + PNT; C (reference)= unprocessed poultry + processed poultry + PNT). We applied methods to 2015–18 National Health and Nutrition Examination Survey data to estimate RM and poultry intake prevalence and amount. We estimated and compared intakes within RM and within poultry methods *via* the NCI Method for individuals ≥2 years old (*n* = 15,038), adjusted for age, sex, and race/Hispanic origin. We compared the population percentage that exceeded age- and sex-specific RM and poultry allotments from the Dietary Guidelines for Americans recommended eating patterns. The percent that consumed RM ranged from 47 ± 1.2% to 75 ± 0.8% across methods and mean amount ranged from 10.5 ± 0.28 to 18.2 ± 0.35 lean oz-equivalents/week; 38 ± 1.2% to 71 ± 0.7% and 9.8 ± 0.35 to 13.3 ± 0.35 lean oz-equivalents/week across poultry methods. Estimates for higher, but not lower, intake percentiles differed across RM methods. Compared to the reference, Method 1 was ≥3.0 oz-equivalents/week lower from 20th-70th percentiles, ≥6.0 oz-equivalents/week lower from 75th-90th percentiles, and ≥9.0 oz-equivalents/week lower for the 95th percentile. Method 4, but not Method 3, was ≥3.0 oz-equivalents/week higher than the reference from 50 to 95th percentiles. The population percentage that exceeded allotments was 27 ± 1.8% lower for Method 1, 9 ± 0.8% higher for Method 3, and 14 ± 0.9% higher for Method 4 compared to the reference. Differences were less pronounced for poultry. Our analysis quantifies the magnitude of bias introduced by heterogeneous meat food group methodology. Explicit descriptions of meat food groups are important for development of dietary recommendations to ensure that research studies are compared appropriately.

## Introduction

Dietary guidance in the U.S. emphasizes adoption of food group-based dietary patterns to meet nutrient needs and prevent chronic disease risk (1). Heterogeneity in research questions, study design, sample populations, and dietary assessment methodologies precludes adoption of standardized food groups or food group lexicons (2) by nutrition researchers. Dietary assessment tools, such as food frequency questionnaires and 24-h dietary recalls, suit different research purposes and collect varying levels of detailed data on each reported food group (3). The level of detail of the dietary assessment tool(s) employed dictates how food groups can be subsequently operationalized. Even with comprehensive data collected at the individual food level, there is a lack of standardized definitions of food groups across public health and research organizations to guide researchers and the public (4, 5). These two factors contribute heterogeneity to how researchers operationalize food groups across research studies, influencing scientists' and policy makers' ability to collate and translate research into food-based dietary pattern recommendations.

The 2015–20 and 2020–25 Dietary Guidelines for Americans (DGA) scientific advisory committees noted that heterogeneity in food groups was most prominent in research about meat intake and chronic disease risk (6, 7). A systematic review showed that meat terminology used throughout chronic disease literature as well as the foods included within meat food groups differed within and between observational and experimental nutrition research studies (8). A challenge in assessing meat subgroups is that dietary assessment tools and database do not disaggregate processed meat into processed red meat and processed poultry inhibiting researchers' ability to create accurate and detailed red meat and poultry food groups. For example, this has led to processed red meat and processed poultry being omitted, thus researchers estimate intakes of unprocessed red meat and unprocessed poultry only. Or researchers have grouped all processed meat, inclusive of processed red meat and processed poultry, with unprocessed red meat, hence the “red and processed meat” food group commonly used in the literature (8). Further, about 25% of researchers don't include any description of how red meat and poultry food groups are operationalized which provide no indication of potential misclassification (9–11). One would hypothesize that variations in analytical decisions of how to operationalize food groups would meaningfully influence intake estimates because each method represents a distinct and unique food group. Therefore, the objective of this analysis was to assess how intakes of red meat and poultry and the proportion of the population below, within, and above allotments from the 2020–25 DGA recommended eating patterns differs based on the method used to operationalize red meat and poultry food groups. This analysis will aid understanding of the degree to which misclassification within meat food groups influences population-level intakes.

## Methods

### Study Design

We used data from the 2015–16 and 2017–18 National Health and Nutrition Examination Survey (NHANES) which is conducted by the U.S. Centers for Disease Control and Prevention's National Center for Health Statistics (NCHS). NHANES uses a multistage, complex, probability sample to release health and nutrition data every 2 years that is representative of the non-institutionalized U.S. population (12). Participants are recruited for a household interview and a physical examination conducted in the NHANES Mobile Examination Centers (MEC). Survey design and analytical weighting procedures are described in detail previously (13).

### Ethics

All NHANES protocols are approved by the NCHS Research Ethics Review Board (14, 15). Participants aged ≥18 years provide written consent, and written consent is provided by a parent or guardian for those aged <18 years. Additional assent is obtained for those 7–17 years.

### Demographic Data Collection

Self-reported demographic data are collected during the at-home interview (16, 17). Demographic variables relevant to this analysis were age (≥2 years old), gender (male, female), race and Hispanic origin (Non-Hispanic White, Non-Hispanic-Black, Non-Hispanic Asian, and Hispanic), family income to poverty ratio (PIR; ≤ or >130% which is the cut off for the Supplemental Nutrition Assistance Program), educational attainment for participants 19 years old (high school or less, more than high school), and head of household educational attainment for participants 2–18 years old (high school or less and more than high school).

### Dietary Intake Data Collection

Dietary data are available from NHANES *via* a joint effort between NCHS and the US Department of Agriculture (USDA) and are referred to as the What We Eat In America (WWEIA) (18) component. Self-reported dietary data are collected in the MEC *via* trained interview administered 24-h recalls using the USDA's computer assisted Automated Multiple Pass Method (18). Participants are asked to recall what foods, beverages, and dietary supplements they consumed the prior day and the amount they consumed. Participants ≥12 years old completed the 24-h dietary recall on their own, participants 6–11 years old were assisted by a parent or guardian, and participants ≤ 5 years old had a parent or guardian proxy complete the 24-h recall. A second 24-h dietary recall is conducted *via* telephone 3–10 days later. Each food and beverage reported in a 24-h dietary recall is coded to correspond to a food code in the USDA's Food and Nutrient Database for Dietary Studies (FNDDS) (19). Each food code subsequently links to the Food Patterns Equivalents Database (FPED) which disaggregates food code components into servings sizes [ounce-equivalents (oz.-eq), cup equivalents, teaspoon equivalents, or grams] of 37 distinct food pattern components (7 of which are related to meat intake) used to model the food patterns recommended in the DGA (20). Each food code is also linked to a WWEIA food category and subcategory which describes the food code “as consumed,” e.g., “bacon” or “burgers” (21).

### Red Meat and Poultry Food Groups

There is heterogeneity in food group definitions across public health and research organizations (4, 5). Therefore, it is important for us to describe the definitions used for this analysis. No public health or research organization defines and describes all meat food groups needed for our purposes, so we relied on three resources: the FPED (2), the DGA (1, 22), and the American Meat Science Association (AMSA) Lexicon (23). Overall, the term “meat” refers to “skeletal muscle and associated tissues derived from mammalian, avian, reptilian, amphibian, and aquatic species harvested for human consumption” (23). The FPED defines (1) “meat,” i.e., red meat, as “beef, veal, pork, lamb, and game meat; excludes organ meat and cured meat;” (2) poultry as “chicken, turkey, Cornish hens, duck, goose, quail, and pheasant (game birds); excludes organ meat and “cured meat;” and (3) cured meat as “frankfurters, sausages, corned beef, cured ham and luncheon meat that are made from beef, pork, or poultry.” The “cured meat” FPED variable encompasses most processed meat consumed in the US (2). Therefore, the term “processed” rather than “cured” will be used throughout the manuscript to be consistent with DGA terminology (22, 24). The AMSA Lexicon was used to identify additional types of processed meat, other than cured, that could be estimated using FNDDS data. This resulted in additionally including chicken patties, nuggets, and tenders as processed poultry products because they are considered further processed by AMSA and are reasonably estimated in FNDDS using the WWEIA categories. By default, meats that are not processed will be referred to as “unprocessed.” The gram weight of solid fats present in meat above 2.63 grams is allocated to the solid fat FPED gram weight rather than meat (21, 22). Therefore, all red meat and poultry food groups operationalized in our analysis are in lean meat ounce-equivalents (oz-eq).

**Figure 1:**
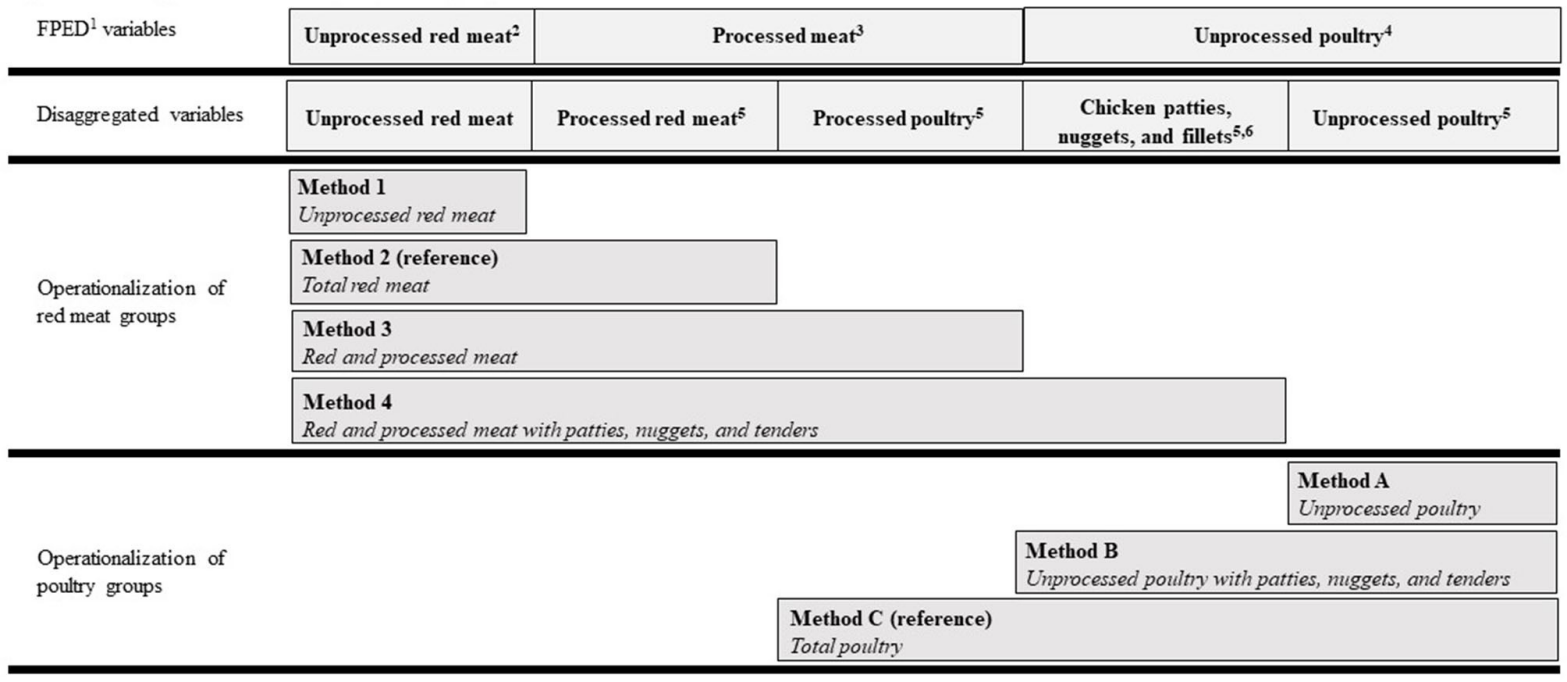
Ontology of red meat and poultry food group methods used in nutrition research. ^1^The US Department of Agriculture's Food Patterns Equivalents Database, available here: https://www.ars.usda.gov/northeast-area/beltsville-md-bhnrc/beltsville-human-nutrition-research-center/food-surveys-research-group/. ^2^“Meat” FPED (PF_meat), defined as beef, veal, pork, lamb, and game meat; excludes organ meat and cured meat. ^3^“Cured meat” FPED (PF_curedmeat), defined as frankfurters, sausages, corned beef, cured ham and luncheon meat that are made from beef, pork, or poultry. ^4^“Poultry” FPED (PF_poult), defined as chicken, turkey, Cornish hens, duck, goose, quail, and pheasant (game birds); excludes organ meat and cured meat. ^5^Disaggregated from FPED using the Processed Meat Categories SAS program from the US National Cancer Institute, available here: https://epi.grants.cancer.gov/ProcessedMeatCategories/. ^6^Considered “further processed” by American Meat Science Association Lexicon, available here: https://dl.sciencesocieties.org/publications/mmb/articles/2/3/1.

We used a systematic review of meat terminology to build an ontology of common methods in which “red meat” and “poultry” food groups were operationalized by researchers in the nutrition and chronic disease literature (8). A challenge in assessing red meat and poultry intake is that dietary assessment tools and available dietary databases do not disaggregate processed meat groups into processed red meat and processed poultry. Therefore, researchers generally defaulted to one of the following described methodological decisions when assessing red meat and/or poultry intake (8). First, researchers may exclude processed meat completely and assess only unprocessed red meat or unprocessed poultry (2, 25, 26) which underestimates true red meat and poultry intakes. Second, researchers may classify all processed meat as processed red meat, i.e., “red and processed meat” (27–29), which overestimates red meat and underestimates poultry. A third option is to disaggregate processed meat into processed red meat and processed poultry and reaggregate with unprocessed red meat and unprocessed poultry, respectively. Yet, dietary assessment methods and available dietary databases rarely allow for this option. For our analysis, we used the NCI Processed Meat Categories SAS program (30) to disaggregate the processed meat FPED variable into processed red meat and processed poultry. This program also disaggregates chicken patties, nuggets, and tenders from the unprocessed poultry FPED because these are considered processed by some definitions (23). In brief, this program text-mines descriptive data for all food codes in FNDDS that contain a processed meat FPED component. The details of the code are previously described (30). In summary, for this analysis we compared four methods of operationalizing a red meat food group and three methods of operationalizing a poultry food group which are described in Figure 1.

### Red Meat and Poultry Allotments

To calculate allotment ranges, we first determined age- and sex-specific energy intake ranges (from sedentary to active) using the Institute of Medicine's estimated energy requirements for each population subgroup 2020–25 DGA (1). Those energy intake ranges were corresponded to red meat and poultry allotment amounts modeled in the recommended food patterns from the 2020–25 DGA (31) (Supplementary Table 1.1). The red meat categories in Figure 1 were compared to the “meat” (i.e., red meat) allotment ranges and the poultry categories were compared to the poultry allotment ranges. Additional details about this analysis are described in the footnotes of Supplementary Table 1.1.

### Analytical Sample

Our analytical sample consists of all participants aged ≥2 years old who participated in the MEC examination of the 2015–16 and 2017–18 NHANES cycles (*n* = 17,945). Participants were excluded if they did not provide at least one dietary recall deemed reliable by NCHS data reporting standards (*n* = 2,907) (32), resulting in a final analytical sample of 15,038 participants. Of those participants, 84.4% had a reliable second dietary recall from which the data were used in statistical modeling of usual intakes described below. Unweighted examination response rates for participants in this age range were 58.7% in 2015–16 and 48.8% in 2017–18 (33). Enhanced weighting adjustments are applied by NCHS to limit potential response rate biases for population subgroups (34).

### Statistical Analysis

Population characteristics and intake prevalence (i.e., the percent of the population who reported consuming red meat or poultry) were estimated *via* survey commands in SAS Version 9.4 (SAS Institute Inc. Cary, NC, USA). For each method described in Figure 1, we used The NCI Method (35, 36) to estimate usual intake (1) distributions of red meat and poultry and (2) proportions of the population whose intakes were below, within, or above red meat and poultry allotment ranges from the 2020–25 DGA recommended eating patterns. We chose the two-part model (i.e., for episodic food consumption) because >10% of the population had zero intakes of all red meat and poultry food groups listed in Figure 1 (37). Integerized balanced repeated replication (BRR) weights were used to account for the day of the week that the 24-h recall was conducted, differential weighting for subpopulations, and the multistage complex sampling design of NHANES (36). We calculated 32 BRR weights by all 60 post-stratification combinations of age, sex, and race/ethnicity consistent with the NHANES sampling methods and used 0.3 for the Fay method which correlates with a perturbation factor of 70% (38). Weekly red meat and poultry allotments from the 2020-25 DGA were divided by 7 to be incorporated into The NCI Method SAS macros. The results are presented on a weekly basis in which estimated means and standard errors were multiplied back by 7 to be consistent with the DGA food patterns (1). Estimates were adjusted for age, gender, and race/Hispanic origin.

We then conducted pairwise comparisons to investigate differences in estimates within red meat food group methods and within poultry food group methods to assess how operationalization of each method influences population-level estimates. We used *total red meat* (red meat Method 2) and *total poultry* (poultry Method 3) as the reference method because this is likely the estimate that is most representative of true red meat and poultry intakes. We will highlight total red meat and total poultry intake estimates throughout the results, as intake estimates of these two food groups are a novel contribution to the literature. Due to the large sample size and increased precision *via* measurement error correction *via* usual intake modeling, even the most conservative Bonferroni correction (*P* < 0.00007) resulted in most comparisons being statistically significant. Therefore, we will highlight effect sizes rather than the Bonferroni corrected *P* values throughout the results (39). Comparing intake amounts, we note meaningful effect sizes between methods as a difference of ≥3, ≥6, or ≥9 lean oz-eq/week. Three lean oz-eq is a recommended serving size of meat, so these effect sizes can be interpreted as a difference of 1, 2, or 3 servings/week between methods. When comparing the percent of population below, within, or above allotment ranges, we note meaningful effect sizes between methods as a difference of ≥10% and ≥20%. All analyses accounted for the complex survey design of NHANES and were weighted using day one dietary intake sample weights to account for oversampling, non-response, and post-stratification.

## Results

### Prevalence of Consumption and Mean Intake

Our sample is nationally representative of the U.S. population aged ≥2 years old and is described in Table 1. Total red meat (i.e., unprocessed and processed red meat) is consumed in 70 ± 0.8% of the population, with a mean total red meat intake of 14.0 ± 0.35 lean oz-eq/week. Total poultry (i.e., unprocessed and processed poultry) is consumed in 71 ± 0.7% of the population, with a mean total poultry intake of 13.3 ± 0.35 lean oz-eq/week. Individuals <19 years old are consuming more total poultry than red meat, and individuals ≥19 years old consume more total red meat than total poultry (Table 2).

**Table 1:** Demographic characteristics of individuals from a representative sample of the US population.

**Characteristic**	**Age 2–18 years**	**Age ≥19 years**	**Age ≥2 years**
Total	5,037	10,001	15,038
Sex			
Male	2,508 (51%)	4,850 (48%)	7,358 (49%)
Female	2,529 (49%)	5,151 (52%)	7,680 (51%)
Race and Hispanic origin			
Non-Hispanic White	1,566 (51%)	3,467 (63%)	5,033 (60%)
Non-Hispanic Black	1,135 (13%)	2,238 (11%)	3,373 (12%)
Non-Hispanic Asian	438 (4%)	1,172 (6%)	1,610 (6%)
Hispanic	1,477 (24%)	2,688 (16%)	4,165 (18%)
**PIR**			
<130%	1,834 (31%)	2,723 (21%)	4,557 (24%)
≥130%	2,772 (69%)	6,210 (79%)	8,982 (76%)
Educational attainment			
High school or less	1,645 (71%)	4,295 (37%)	5,940 (41%)
More than high school	492 (29%)	5,453 (63%)	5,945 (59%)

*Data presented as unweighted number of participants in each sample strata and weighted percent of population is presented in parentheses. Data source: US Centers for Disease Control and Prevention/National Center for Health Statistics, NHANES 2015–2018*.

**Table 2:** Prevalence and amount of self-reported intake of red meat and poultry in the US estimated *via* different methods.

**Characteristic**	**Age 2–18 years *n* = 5,037**	**Age ≥19 years *n* = 10,001**	**Age ≥2 years *n* = 15,038**
**Intake prevalence of red meat (%)**			
Method 1: Unprocessed red meat	40 ± 1.3	49 ± 1.3	47 ± 1.2
Method 2: Total red meat	67 ± 1.3	70 ± 0.8	70 ± 0.8
Method 3: Red and processed meat	70 ± 1.3	73 ± 0.8	72 ± 0.8
Method 4: Red and processed meat with patties, nuggets, and tenders	76 ± 1.2	75 ± 0.8	75 ± 0.8
**Mean intake amount of red meat (lean oz-eq/week)**			
Method 1: Unprocessed red meat	7.0 ± 0.35	11.2 ± 0.35	10.5 ± 0.28
Method 2: Total red meat	9.8 ± 0.35	15.4 ± 0.35	14.0 ± 0.35
Method 3: Red and processed meat	12.6 ± 0.35	18.2 ± 0.35	16.8 ± 0.35
Method 4: Red and processed meat with patties, nuggets, and tenders	14.7 ± 0.47	18.9 ± 0.42	18.2 ± 0.35
**Intake prevalence of poultry groups (%)**			
Method A: Unprocessed poultry	32 ± 1.3	40 ± 1.1	38 ± 1.0
Method B: Unprocessed poultry with patties, nuggets, and tenders	43 ± 1.7	43 ± 1.1	43 ± 1.0
Method C: Total poultry	75 ± 1.2	70 ± 0.8	71 ± 0.7
**Mean intake amount of red meat (lean oz-eq/week)**			
Method A: Unprocessed poultry	7.0 ± 0.42	10.5 ± 0.35	9.8 ± 0.35
Method B: Unprocessed poultry with patties, nuggets, and tenders	9.1 ± 0.49	11.2 ± 0.35	10.5 ± 0.35
Method C: Total poultry	11.2 ± 0.56	14.0 ± 0.35	13.3 ± 0.35

*Weighted mean ± standard error of the mean. See Figure 1 for ontology of red meat and poultry method. Data source: US Centers for Disease Control and Prevention/National Center for Health Statistics, NHANES 2015–2018, day 1 dietary recall data*.

The prevalence of those ≥2 years old reporting consumption of red meat ranges from 47 ± 1.2% to 75 ± 0.8% of the population and mean intake amounts range from 10.5 ± 0.28 to 18.2 ± 0.35 lean oz-eq/week (Table 2), depending on which method is operationalized (see ontology in Figure 1). The prevalence of those ≥2 years old reporting poultry intake ranges from 38 ± 1.0% to 71 ± 0.7% and mean intake amounts range from 9.8 ± 0.35 to 13.3 ± 0.35 lean oz-eq/week (Table 2) depending on which method is operationalized (see ontology in Figure 1). Weekly mean intake estimates for each age and sex subgroup are shown in Supplementary Tables 1.3, 1.4.

#### Red Meat

**Figure 2:**
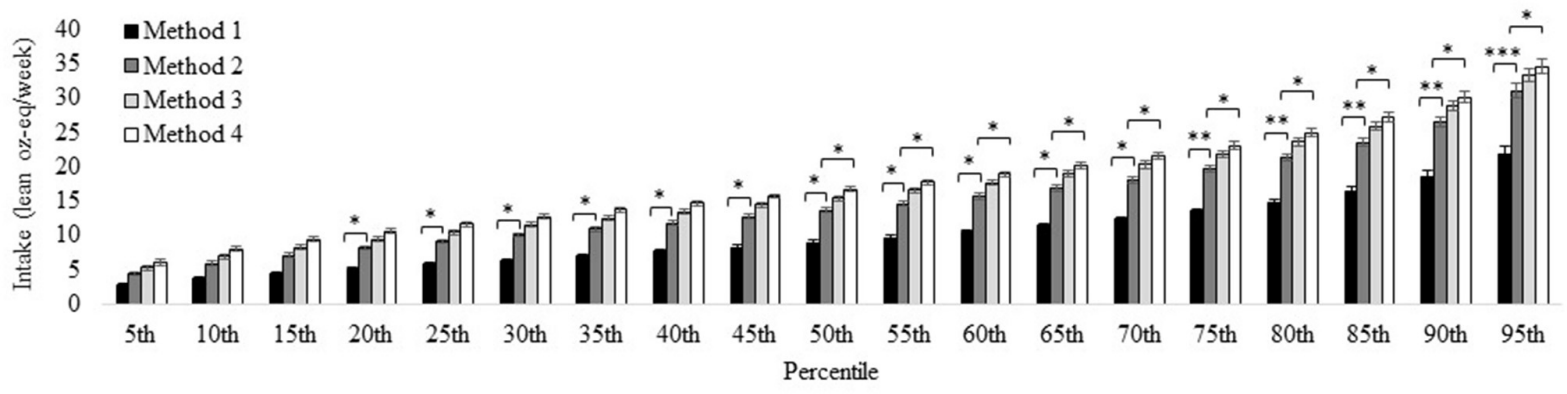
Distribution of red meat intake for the U.S. population aged ≥2 years estimated *via* different methods. Method 1: Unprocessed red meat, includes beef, veal, pork, lamb, and game meat; excludes organ meat and processed meat. Method 2 (reference): Total red meat, includes unprocessed red meat and processed red meat. Method 3: Red and processed meat, includes unprocessed red meat, processed red meat, and processed poultry. Method 4: Red and processed meat, additionally including chicken patties, nuggets, and tenders. See Figure 1 for further descriptions of each method. Results are shown as mean ± SEM within each percentile estimated *via* the NCI Method for usual dietary intakes. *Difference from Method 1 estimate (effect size) is ≥ 3.0 oz-eq per week; **difference from Method 1estimate is ≥ 6.0 oz-eq servings per week; ***difference from Method 1 estimate is ≥ 9.0 oz-eq per week. Data source: US Centers for Disease Control and Prevention/National Center for Health Statistics, NHANES 2015-2018, day 1 and 2 dietary recall data.

Intake of total red meat (Method 2, and the reference for all comparisons) for individuals aged ≥2 years was 13.6 ± 0.32 lean oz-eq/week at the 50th percentile and 31.0 ± 1.04 lean oz-eq/week at the 95th percentile. Intake increased from Method 1 to 2 (i.e., the progression from unprocessed red meat to total red meat which additionally included processed red meat) by ≥3.0 lean oz-eq/week from the 20–70th percentile, by ≥6.0 lean oz-eq/week from the 75–90th percentile, and by ≥9.0 lean oz-eq/week for the 95th percentile (Figure 2). The differences between intakes of Method 1 (unprocessed red meat) and Method 2 (total red meat) were largest at the higher end of the intake distribution for males of all age categories. Intake estimates using Method 2 vs. Method 3 (i.e., the progression from total red meat to “red and processed meat” which additionally includes processed poultry) were within one 3 lean oz-eq serving/week across the distribution and for each sex and age subgroup. Intake estimates using Method 4 (i.e., additional inclusion of chicken patties, nuggets, and tenders in “red and processed meat”) from the 50th (16.7 ± 0.34 lean oz-eq/week) to 95th (34.6 ± 1.05 lean oz-eq/week) percentile differed from Method 2 (total red meat) by ≥3.0 lean oz-eq/week. See Supplementary Tables 1.5, 1.6 for age- and sex-specific intakes using each red meat method.

#### Poultry

**Figure 3:**
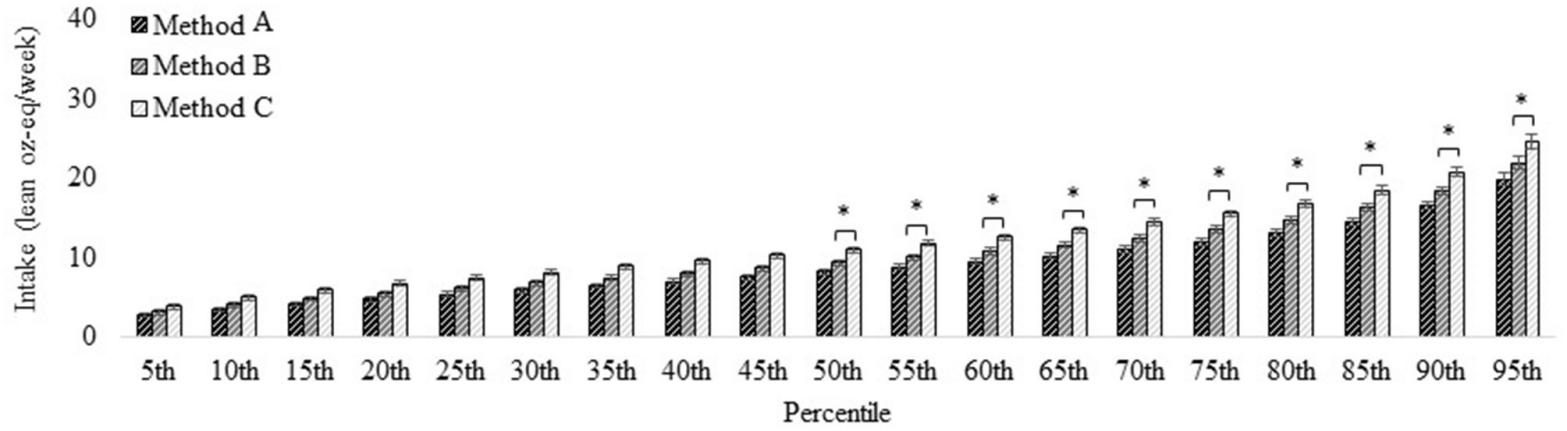
Distribution of poultry intake for the U.S. population aged ≥2 years estimated *via* different methods. Method A: Unprocessed poultry, includes chicken, turkey, Cornish hens, duck, goose, quail, and pheasant (game birds); excludes organ meat and cured meat; additionally excludes chicken patties, nuggets, and tenders. Method B: Unprocessed poultry, includes chicken, turkey, Cornish hens, duck, goose, quail, and pheasant (game birds); excludes organ meat and cured meat and includes chicken patties, nuggets, and tenders. Method C (reference): Total poultry, includes unprocessed poultry, processed poultry, and chicken patties, nuggets, and tenders. See Figure 1 for further descriptions of each method. Results are shown as mean ± SEM within each percentile estimated *via* the NCI Method for usual dietary intakes. *Difference from Method 1 estimate (effect size) is ≥ 3.0 oz-eq per week. Data source: US Centers for Disease Control and Prevention/National Center for Health Statistics, NHANES 2015–2018, day 1 and 2 dietary recall data.

Intake of total poultry (Method C and the reference for all comparisons) for individuals aged ≥2 years was 11.0 ± 0.37 lean oz-eq/week at the 50th percentile and 24.6 ± 0.86 lean oz-eq/week at the 95th percentile. Estimates using Method A were ≥3.0 lean oz-eq/week less than Method C from the 50–95th percentile (i.e., the progression from unprocessed poultry, excluding chicken patties, nuggets, and tenders to total poultry inclusive of chicken patties, nuggets, and tenders as well as processed poultry products). Estimates using Method B did not differ from Method C (Figure 3), in which the difference between the two methods is the additional inclusion of processed poultry products in Method C (total poultry). Differences between methods were similar across all age and sex subgroups (Supplementary Tables 1.7, 1.8).

### Comparison of Intakes to Allotment Ranges From the 2020–25 DGA Eating Patterns

#### Red Meat

We compared intake amounts of individuals ≥2 years old to the corresponding red meat allotment ranges from the 2020–25 DGA recommended eating patterns. For total red meat (Method 2 and the reference for all comparisons), 35 ± 2.0% of the population was below, 20 ± 1.1% was within, and 45 ± 1.8% was above their age- and sex-specific red meat allotment ranges.

**Figure 4:**
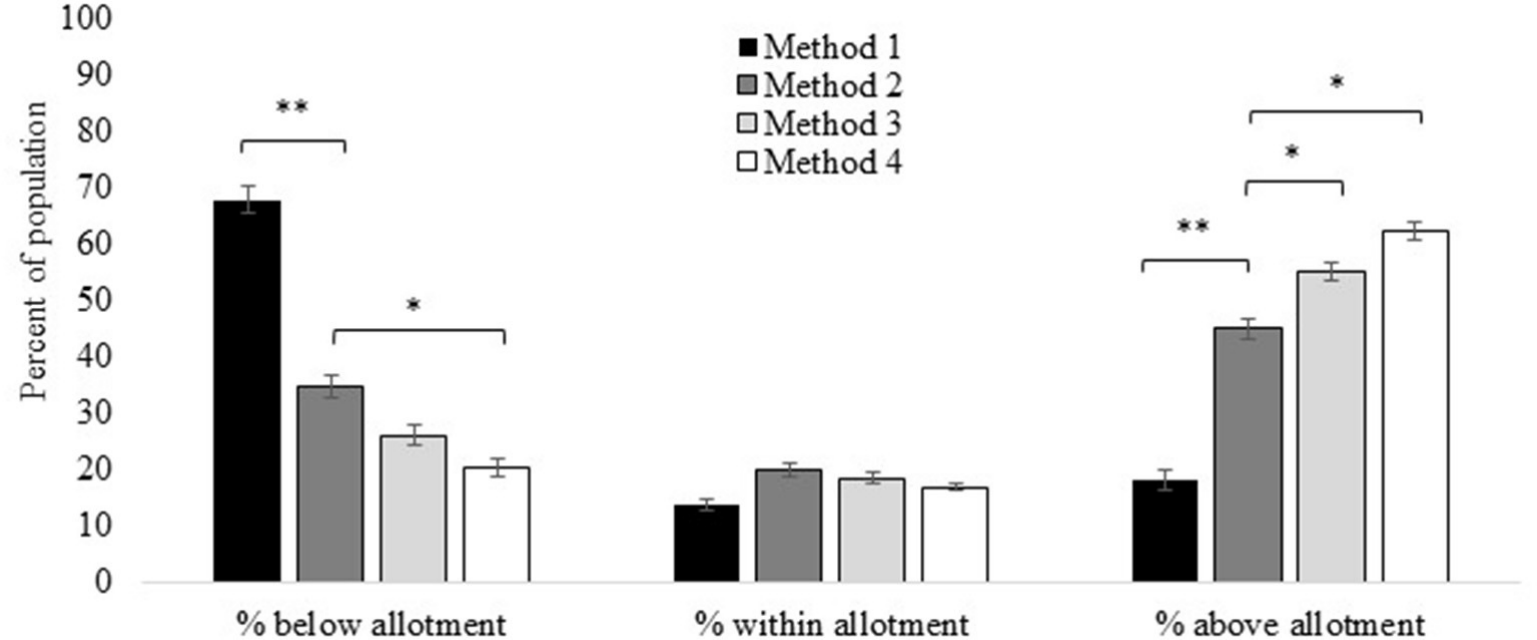
Red meat intakes compared to allotment ranges in the US 2020–25 Dietary Guidelines for Americans recommended eating patterns estimated *via* different methods. Method 1: Unprocessed red meat, includes beef, veal, pork, lamb, and game meat; excludes organ meat and processed meat. Method 2 (reference): Total red meat, includes unprocessed red meat and processed red meat. Method 3: Red and processed meat, includes unprocessed red meat, processed red meat, and processed poultry. Method 4: Red and processed meat, additionally including chicken patties, nuggets, and tenders. See Figure 1 for further descriptions of each method. Results are shown as mean ± SEM *via* the NCI Method for usual dietary intakes. *Difference from Method 1 estimate (effect size) is ≥10%; **difference from Method 1 estimate is ≥20%. Data source: US Centers for Disease Control and Prevention/National Center for Health Statistics, NHANES 2015-2018, day 1 and 2 dietary recall data.

The percentage of the population that had intakes below their allotment range decreased by 33 ± 2.6% from Method 1 to Method 2 (i.e., the progression from unprocessed red meat to total red meat which includes processed red meat; Figure 4; Supplementary Table 1.9). The percentage was similar between Method 3 and Method 2 (i.e., the progression from total red meat to “red and processed meat” which additionally includes processed poultry) but was 14 ± 0.9% lower when using Method 4 (i.e., additional inclusion of chicken patties, nuggets, and tenders in “red and processed meat”) vs. Method 2 (total red meat). The percent of the population whose intakes were within their age- and sex-specific allotment range did not differ by ≥10% based on which red meat method was used (Figure 4; Supplementary Table 1.10). The percent of the population who had intakes above their age- and sex-specific allotment was 27 ± 1.8% lower when using Method 1 (unprocessed red meat) vs. Method 2 (total red meat), was 10 ± 0.7% higher when using Method 3 (red and processed meat) vs. Method 2 (total red meat), and 17 ± 1.1% higher when using Method 4 (red and processed meat plus chicken patties, nuggets, and tenders) vs. Method 2 (total red meat).

**Figure 5:**
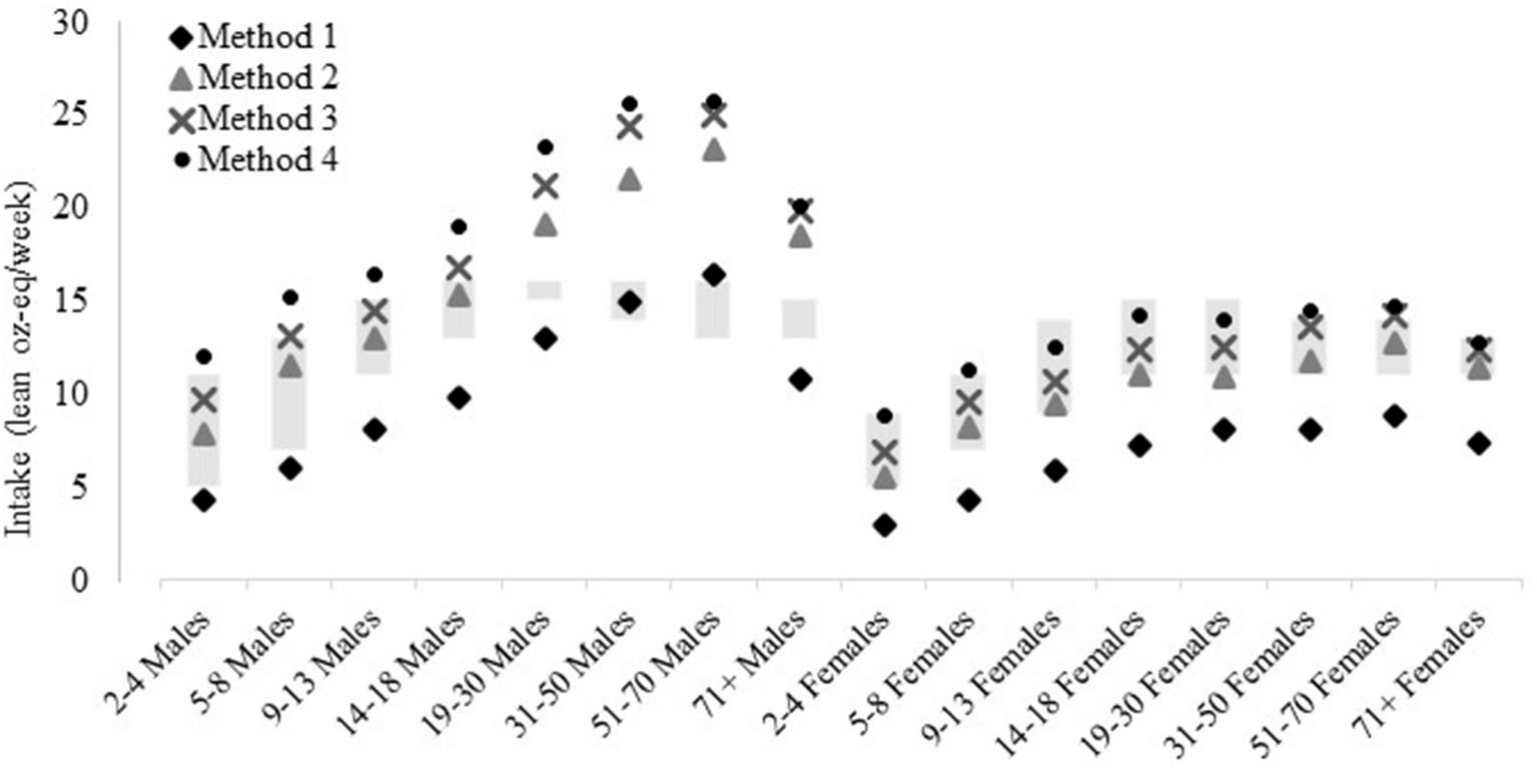
Red meat intakes compared to allotment ranges in the US 2020-25 Dietary Guidelines for Americans recommended eating patterns, by age and sex, estimated *via* different methods. Method 1: Unprocessed red meat, includes beef, veal, pork, lamb, and game meat; excludes organ meat and processed meat. Method 2 (reference): Total red meat, includes unprocessed red meat and processed red meat. Method 3: Red and processed meat, includes unprocessed red meat, processed red meat, and processed poultry. Method 4: Red and processed meat, additionally including chicken patties, nuggets, and tenders. See Figure 1 for further descriptions of each method. Results are shown as mean estimated *via* the NCI Method for usual dietary intakes. Data source: US Centers for Disease Control and Prevention/National Center for Health Statistics, NHANES 2015-2018, day 1 and 2 dietary recall data.

Most age and sex subgroups consumed amounts of total red meat (Method 2; reference) that were within red meat allotment ranges from the 2020–25 DGA eating patterns (Figure 5). The exceptions are males aged 19–30, 31–50, 51–70, and 71+ years who consumed amounts above the allotment ranges. Intakes were below the age- and sex-specific red meat allotment ranges when using Method 1 (unprocessed red meat). The exceptions were males aged 31–50 whose mean intakes were within the allotment range, and males aged 51–70 whose intakes were just above the allotment range. Results were similar when using Method 3 (red and processed meat) and Method 2 (total red meat), except that the inclusion of processed poultry in Method 3 pushed intakes of males aged 14–18 years above allotment ranges. When chicken patties, nuggets, and filets were additionally included in Method 4, mean intakes of males aged 2–4, 5–8, and 9–13 years were pushed above allotment ranges. See Supplementary Table 1.3 for age- and sex-specific mean intakes for each red meat method.

#### Poultry

**Figure 6:**
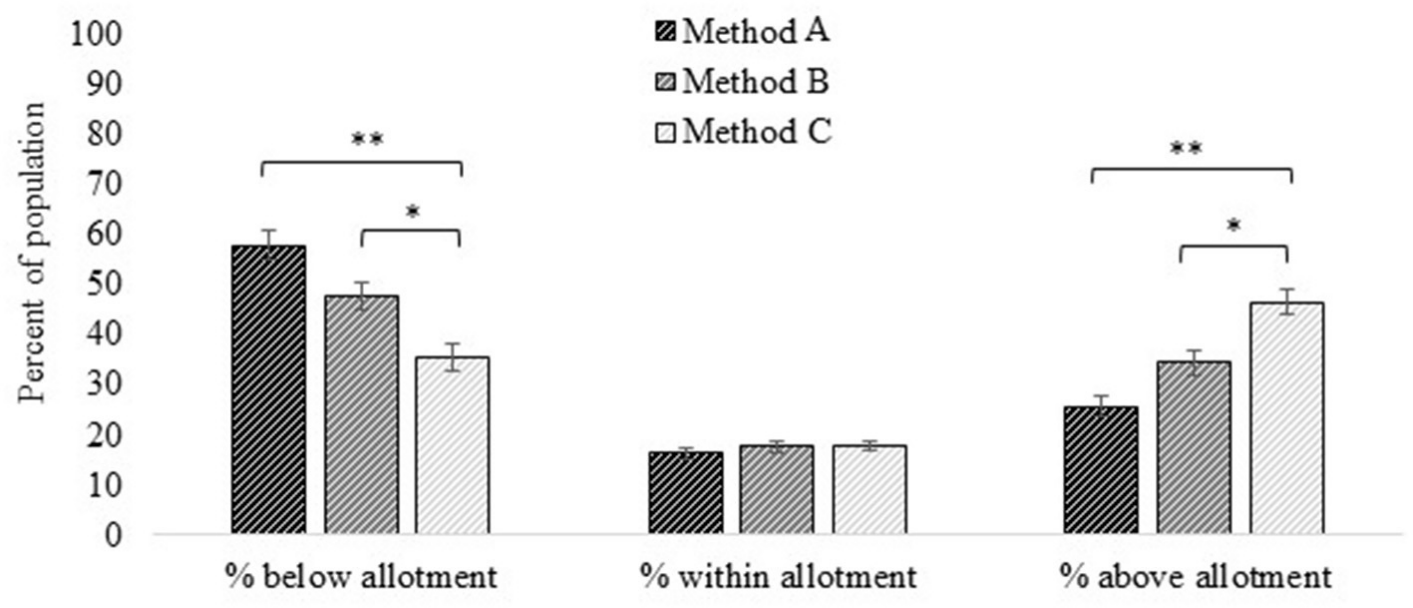
Poultry intakes compared to allotment ranges in the US 2020–25 Dietary Guidelines for Americans recommended eating patterns estimated *via* different methods. Method A: Unprocessed poultry, includes chicken, turkey, Cornish hens, duck, goose, quail, and pheasant (game birds); excludes organ meat and cured meat; additionally excludes chicken patties, nuggets, and tenders. Method B: Unprocessed poultry, includes chicken, turkey, Cornish hens, duck, goose, quail, and pheasant (game birds); excludes organ meat and cured meat and includes chicken patties, nuggets, and tenders. Method C (reference): Total poultry, includes unprocessed poultry, processed poultry, and chicken patties, nuggets, and tenders. See Figure 1 for further descriptions of each method. Results are shown as mean ± SEM within each percentile estimated *via* the NCI Method for usual dietary intakes. *Difference from Method 1 estimate (effect size) is ≥10%; **difference from Method 1 estimate is ≥20%. Data source: US Centers for Disease Control and Prevention/National Center for Health Statistics, NHANES 2015-2018, day 1 and 2 dietary recall data.

We compared intake of individuals ≥2 years old to the corresponding poultry allotment ranges from the 2020–25 DGA recommended eating patterns. Using total poultry (Method C and reference for all comparisons), 36 ± 2.6% of the population was below, 18 ± 0.9% was within, and 47 ± 2.6% was above age- and sex-specific allotment ranges (Figure 6). The percentage of the population that had intakes below their allotment range was 12 ± 1.1% higher with Method B (unprocessed poultry, including chicken tenders, nuggets, and patties) than Method C (total poultry). The percentage of the population that had intakes below their allotment range was ≥20% higher using Method A (unprocessed poultry, excluding chicken tenders, nuggets, and patties) vs. Method C (Figure 6; Supplementary Table 1.12). There was no difference between poultry methods for the percent within allotment ranges, except a lower percent of males and females aged 5–8 years (Supplementary Table 1.13) were within allotment ranges when using Method A vs. Method C. Method C differed from Method B by 12 ± 0.8% and differed from Method A by ≥20% when estimating the percent above the allotment range. The largest differences were between Method A and Method C for the younger age and sex subgroups (Supplementary Table 1.14).

**Figure 7:**
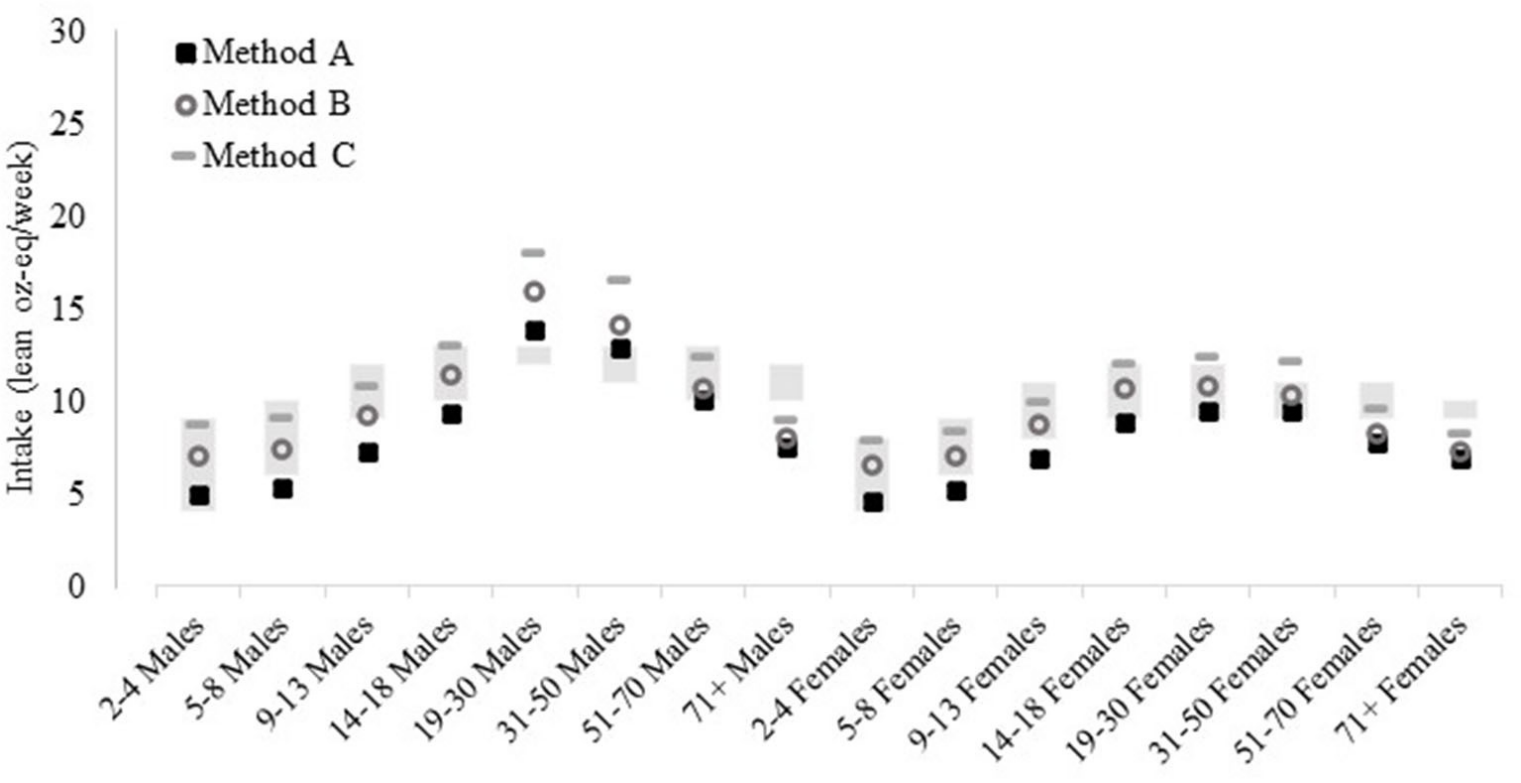
Poultry intakes estimated by various poultry food group methods compared to allotment ranges in the US 2020–25 Dietary Guidelines for Americans recommended eating patterns, by age and sex, estimated *via* different methods. Method A: Unprocessed poultry, includes chicken, turkey, Cornish hens, duck, goose, quail, and pheasant (game birds); excludes organ meat and cured meat; additionally excludes chicken patties, nuggets, and tenders. Method B: Unprocessed poultry, includes chicken, turkey, Cornish hens, duck, goose, quail, and pheasant (game birds); excludes organ meat and cured meat and includes chicken patties, nuggets, and tenders. Method C (reference): Total poultry, includes unprocessed poultry, processed poultry, and chicken patties, nuggets, and tenders. See Figure 1 for further descriptions of each method. Results are shown as mean estimated *via* the NCI Method for usual dietary intakes. Data source: US Centers for Disease Control and Prevention/National Center for Health Statistics, NHANES 2015–2018, day 1 and 2 dietary recall data.

Mean intake of total poultry (Method C) is within the age- and sex-specific poultry allotment ranges from the DGA recommended eating patterns for almost all age and sex subgroups (Figure 7). The exceptions were males aged 19–30 and 31–50 years and females aged 19–30 and 31–50 years who were above their allotment ranges and males and females aged ≥71 years who were below allotment ranges. A similar pattern was seen for Method B (unprocessed poultry including chicken tenders, nuggets, and patties), but females aged 19–30 and 31–50 years were within allotment range and females 51–70 years were below with Method B. When chicken patties, nuggets, and tenders are further excluded in Method A, males and females aged 5–8, 9–13, and 14–18 were below allotment ranges.

## Discussion

Heterogeneity in meat terminology and operationalization of meat food groups represents a methodological challenge in elucidating chronic disease implications of meat consumption (4, 6–8). The objective of this analysis was to demonstrate and quantify an example of how heterogeneity in meat food groups impacts population-level estimates of red meat and poultry intakes. Our results were supportive of our hypotheses that variation in meat food groups would meaningfully influence population-level intake estimates. In particular, decisions to include or exclude processed products from red meat and poultry food groups meaningfully changed weekly intake estimates of US populations. This methodological decision also meaningfully changed the percent of individuals who are below, within, and above red meat and poultry allotment ranges from the 2020–25 DGA recommended eating patterns. For example, most age and sex subgroups are below the allotment red meat range when processed red meat products are excluded but are within or substantially above (e.g., males aged 19–70 years old) the allotment range when processed red meat products are included. Further, misclassifying processed poultry as processed red meat, i.e., using the broad “red and processed meat” food group, exacerbates these differences. This analysis demonstrated that misclassification of processed meat and how processed meats are defined within meat food groups meaningfully influence population-level intake estimates of red meat and poultry.

In addition to a methodological demonstration, our analysis also provides novel intake estimates of total lean red meat and total lean poultry for the US population. The USDA routinely estimates unprocessed lean red meat and unprocessed lean poultry intake in the US (2, 40). Our estimate of total lean red meat and total lean poultry further incorporated processed products using the NCI's Processed Meat Categories method (30), adding novel insight into meat intake behaviors of the US population. Our estimates of total lean red meat intake were 3.0–9.0 lean oz-eq higher than estimates of unprocessed lean red meat across percentiles of consumption, and intake differences between total lean red lean meat and unprocessed lean red meat increased as consumption amounts increased. This emphasizes the importance of including processed red meat in lean red meat food groups to describe population intakes. The differences in intake between total lean red meat and unprocessed lean red meat were greatest for males aged 19–70 years old, who tend to be the highest consumers of animal-based protein sources in the US (1, 40). Differences across distributions were less pronounced for poultry estimates. Further, our estimates for total lean red meat (14.0 ± 0.35 lean oz-eq/week) and total lean poultry (13.3 ± 0.35 lean oz-eq/week) mean intakes were within one oz-eq/week of one another and had similar prevalence of consumption (~70% of individuals ≥2 yeas reported consumption). Adults ≥19 years consumed ~1.5 lean oz-eq/week more total lean red meat than total poultry, but adolescents <19 years consumed ~1.5 lean oz-eq/week more total poultry than total lean red meat. True estimates of total lean red meat and total poultry are likely higher, and potentially more divergent than 1.5 oz-eq/week, because some red meat products (such as bacon or pork chops) and poultry products (fried chicken or retaining the skin) are higher in fat (41). Future methodological development is needed to additionally include the non-lean gram weight of total red meat and total poultry to obtain more accurate population-level intake estimates. This is of particular relevance because high-fat meats are associated with various chronic disease end points, as summarized during the 2020–25 DGA development process (42).

We compared mean intakes of age and sex population subgroups to the age- and sex-specific red meat and poultry allotment ranges from the 2020–25 DGA recommended eating patterns. The allotment ranges in the recommended eating patterns result from food pattern modeling conducted in the evidence review portion of the DGA process (43). The foods chosen to create a representative composite of “red meat” in the food pattern modeling and resulting allotment ranges include both processed and unprocessed red meat. Similarly, the “poultry” composite contains unprocessed and processed poultry. Based on these models, one would expect that total red meat and total poultry would be the most appropriate food group for comparison to the allotment ranges. Yet, the FPED, which is designed to represent the 37 different components of the recommended eating patterns, does not provide total red meat or total poultry variables. Therefore, there is a disconnect between the food pattern modeling and the available FPEDs. Total lean red meat and total lean poultry can be reasonably estimated by linking the Processed Meat Categories program to the FPED, as we demonstrated in this analysis. Our results showed that most population subgroups consumed total red meat amounts within allotment ranges (except males aged 19–70 who were above allotment ranges by up to 7 lean oz-eq/week). Comparatively, excluding processed red meat, we found that intakes of unprocessed red meat were below red meat allotment ranges for most age and sex subgroups, and within allotment ranges for males 31–70 years old. Therefore, inclusion vs. exclusion of processed red meat when comparing intakes to allotment ranges results in very different conclusions about red meat intake behaviors. Notably, it is the processed portion of total red meat and total poultry that seems to be pushing certain population subgroups beyond their age- and sex-specific allotment ranges. Most population subgroups in the US consume total red meat and total poultry amounts that are within their age- and sex-specific allotment ranges, except that males 19–70 years consume too much red meat and males and females aged 19–50 consume too much poultry.

A push for methodological transparency in food group operationalization needs to be met with greater consensus on how food groups are defined. For example, the 2020–25 DGA do not provide an explicit definition of processed meat, but recommend that meat and poultry be consumed as “fresh, frozen, or canned, and in lean forms (e.g., chicken breast or ground turkey) versus processed meats (e.g., hot dogs, sausages, ham, luncheon meats)” (1). The prior 2015–2020 DGA defined processed meat as “all meat or poultry products preserved by smoking, curing, salting, and/or the addition of chemical preservatives” including “all types of meat or poultry sausages (bologna, frankfurters, luncheon meats and loaves, sandwich spreads, viennas, chorizos, kielbasa, pepperoni, salami, and summer sausages), bacon, smoked or cured ham or pork shoulder, corned beef, pastrami, pig's feet, beef jerky, marinated chicken breasts, and smoked turkey products” (22). Other organizations, such as AMSA, consider chicken nuggets to be processed as well (23). Our results demonstrate that nuances in processed meat definitions are a major driver of heterogeneity in meat food groups. This likely has differential implications across life stages that should be considered in research design. For example, classification of chicken patties, nuggets, and tenders as either unprocessed or processed poultry alters poultry intake estimates by ~2 lean oz-eq/week for males and females aged 5–18 years old, but not other age groups. Consensus on processed meat definitions is also important for public health messaging. Processed meat intake is recognized as a cancer risk factor (44–46) and has also been associated with increased risk for cardiovascular disease and type 2 diabetes (47). Processed meat is generally higher in sodium and saturated fat content than unprocessed meat (4). Also, processed meat is commonly cured and cooked with high heat, which increases concentrations of N-nitroso, heterocyclic amine, and polycyclic aromatic carbons (48). There is also potential for high heme-iron intake to increase risk for disease, although this is debated (49, 50). These nutritive and non-nutritive compounds are proposed mechanistic links between processed meat consumption and some cancer types (51, 52). Public health organizations recommend consumers avoid or limit processed meat intake to reduce risk for chronic diseases (1), particularly cancer (44). Yet, it is difficult to communicate this message to the general public without a standardized definition of which meat products are considered processed and should be avoided.

For our analysis, we created an ontology of commonly used methods of estimating red meat and poultry food groups from a rigorous and comprehensive systematic review of nutrition research (8). We compared four methods of operationalizing red meat groups and three methods of operationalizing poultry groups within one nationally representative sample of the US population. Yet, our ontology is certainly not an exhaustive list of how researchers operationalize these groups. For example, some researchers classify “sandwich meats” as unprocessed red meat, yet most sandwich meats (i.e., deli meats) would be considered processed by publicly available definitions as well as within the FPED (4). A second example is the use of the term “white meat” which researchers may use to describe a variety of poultry-containing food groups, sometimes including pork or lean and fatty fish (8, 53). Further, we used NCI's Processed Meat Categories SAS program to disaggregate processed meat into processed red meat and processed poultry. This program has been shown to potentially overestimate processed red meat and underestimate processed poultry by ~10–15% (30). Therefore, in our analysis, the differences between red meat methods may be overestimated and the differences within poultry methods may be underestimated. Our analysis is strengthened by using 24-h recall data and the USDA's databases which provide a comprehensive and detailed assessment of participant intake. Although not an exhaustive list of red meat and poultry food group methods, our results serve as a demonstrative example of why capturing nuances in food groups is quantifiably important in estimating intakes.

## Conclusion

Unsurprisingly, different methods of operationalizing red meat and poultry food groups resulted in different population-level intake estimates, simply because they are distinct and unique food groups. The current analysis quantifiably demonstrates the magnitude of potential bias induced by heterogeneous meat food group methods and for which meat subtypes and population subgroups the bias may be most prominent. Future research is needed to understand if the degree of misclassification in red meat and poultry food groups is meaningful enough to influence associations between red meat and poultry intakes and chronic disease risk, particularly cancer. Understanding this bias becomes important during development of public health dietary recommendations to ensure that studies can be grouped and compared appropriately. This work highlights that it may not be appropriate to compare studies that exclude processed products to studies that include processed products in red meat and poultry estimates because, as the current results demonstrate, these are distinct food groups in which intake estimates can differ by up to 9 lean oz-eq/week. The ultimate meat food group method employed depends largely on the research question and the dietary assessment tool used. However, promoting clear and transparent descriptions of meat food group terminology and methodology will provide clarity for researchers and public health professionals when creating and disseminating evidence-based public recommendations.

## Data Availability Statement

Publicly available datasets were analyzed in this study. This data can be found here: https://www.cdc.gov/nchs/nhanes/index.htm.

## Ethics Statement

The studies involving human participants were reviewed and approved by the National Center for Health Statistics. Written informed consent to participate in this study was provided by the participants' legal guardian/next of kin.

## Author Contributions

LEO, KAH, and JR designed the research. LEO conducted the research, wrote the paper with editorial assistance from all authors, and has primary responsibility for final content. LEO and RP analyzed the data. All authors have read and approved the final manuscript.

## Conflict of Interest

RP is employed by Information Management Services, Inc. The remaining authors declare that the research was conducted in the absence of any commercial or financial relationships that could be construed as a potential conflict of interest.

## Publisher's Note

All claims expressed in this article are solely those of the authors and do not necessarily represent those of their affiliated organizations, or those of the publisher, the editors and the reviewers. Any product that may be evaluated in this article, or claim that may be made by its manufacturer, is not guaranteed or endorsed by the publisher.
