# Supplementary material for: Heterogeneity in Meat Food Groups Can Meaningfully Alter Population-Level Intake Estimates of Red Meat and Poultry
Source: Front Nutr. 2021 Dec 15;8:778369. doi: 10.3389/fnut.2021.778369 (PMC8714904; doi:10.3389/fnut.2021.778369)
Supplement: Supplementary file 1 [file Data_Sheet_1.docx]

Supplementary Material

# Tables

## Estimated energy requirements and corresponding red meat and poultry allotment ranges from the 2020-2025 Dietary Guidelines for Americans in the US

| **Age** | **Sex** | **Estimated energy requirements for sedentary individuals (kcal)** | **Estimated energy requirements for active individuals (kcal)** | **Corresponding weekly red meat allotment range (oz-eq)** | | **Corresponding weekly poultry allotment range (oz-eq)** | |
| --- | --- | --- | --- | --- | --- | --- | --- |
| 2-4 | Females | 1000-1200 | 1000-1400 | 5 | 9 | 4 | 8 |
| 5-8 | Females | 1200-1400 | 1600-1800 | 7 | 11 | 6 | 9 |
| 9-13 | Females | 1400-1600 | 1800-2200 | 9 | 14 | 8 | 11 |
| 14-18 | Females | 1800 | 2400 | 11 | 15 | 9 | 12 |
| 19-30 | Females | 1800-2000 | 2400 | 11 | 15 | 9 | 12 |
| 31-50 | Females | 1800 | 2200 | 11 | 14 | 9 | 11 |
| 51-70 | Females | 1600 | 2000-2200 | 11 | 14 | 9 | 11 |
| 71+ | Females | 1600 | 2000 | 11 | 13 | 9 | 10 |
| 2-4 | Males | 1000-1200 | 1000-1600 | 5 | 11 | 4 | 9 |
| 5-8 | Males | 1200-1400 | 1600-2000 | 7 | 13 | 6 | 10 |
| 9-13 | Males | 1600-2000 | 2000-2600 | 11 | 15 | 9 | 12 |
| 14-18 | Males | 2000-2400 | 2800-3200 | 13 | 16 | 10 | 13 |
| 19-30 | Males | 2400-2600 | 3000 | 15 | 16 | 12 | 13 |
| 31-50 | Males | 2200-2400 | 2800-3000 | 14 | 16 | 11 | 13 |
| 51-70 | Males | 2000-2200 | 2600-2800 | 13 | 16 | 10 | 13 |
| 71+ | Males | 2000 | 2400-2600 | 13 | 15 | 10 | 12 |

## The estimated energy requirement ranges are from: Institute of Medicine. Dietary Reference Intakes for Energy, Carbohydrate, Fiber, Fat, Fatty Acids, Cholesterol, Protein, and Amino Acids. Washington (DC): The National Academies Press; 2002. The red meat and poultry allotment ranges correspond to the lowest and highest estimated energy intake requirement level of that age/sex group. Allotment ranges are shown here on a weekly basis, as they are described in the Dietary Guidelines for Americans (see Supplemental Table 2) but were incorporated into statistical analyses on a daily basis (divided by 7). This required a detailed breakdown of the protein subgroups per energy intake level which was provided to our research team by the USDA’s Center for Nutrition Policy and Promotion (Supplemental Table 2).

## Additional details and example of how red meat and poultry allotments were calculated to estimate the percent of the population above, within, and below allotment amounts: Using females aged 2-4 years as an example, 5 oz-eq/week of red meat corresponds to the lower range of 1000 kcal/day for sedentary individuals and 9 oz-eq/week of red meat corresponds to the upper range of the 1400 kcal/day for active individuals. Therefore, the red meat allotment ranges incorporated into the analyses for females aged 2-4 years was 5-9 oz-eq per week, or 0.7-1.3 oz-eq per day. We estimated the proportion of females aged 2-4 years whose intakes were below, within, or above 0.7-1.3 oz-eq/day. This is a similar method that was used to inform the 2020-2025 DGA Scientific Advisory Committee.

## Protein lean oz-equivalents per energy intake level used in food pattern modeling for the 2020-2025 Dietary Guidelines for Americans Scientific Report

|  | **Energy intake level (kcal)** | | | | | | | | | | | |
| --- | --- | --- | --- | --- | --- | --- | --- | --- | --- | --- | --- | --- |
|  | **1000** | **1200** | **1400** | **1600** | **1800** | **2000** | **2200** | **2400** | **2600** | **2800** | **3000** | **3200** |
| Meats* | 5 | 7 | 9 | 11 | 11 | 13 | 14 | 15 | 15 | 16 | 16 | 16 |
| Poultry* | 4 | 6 | 8 | 9 | 9 | 10 | 11 | 12 | 12 | 13 | 13 | 13 |
| Fish-Hi n3 | 1 | 1 | 1 | 2 | 2 | 2 | 2 | 2 | 2 | 2 | 2 | 2 |
| Fish-Lo n3 | 2 | 3 | 5 | 6 | 6 | 6 | 7 | 8 | 8 | 8 | 8 | 8 |
| Eggs | 1 | 2 | 2 | 3 | 3 | 3 | 3 | 3 | 3 | 4 | 4 | 4 |
| Soy products | 0 | 0 | 0 | 0 | 0 | 0 | 0 | 1 | 1 | 1 | 1 | 1 |
| Nuts & Seeds | 2 | 2 | 3 | 4 | 4 | 4 | 5 | 5 | 5 | 5 | 5 | 5 |

This information was provided upon request from the Data Analysis Team of the 2020 Dietary Guidelines Advisory Committee. These allotment amounts were the same in the 2015-20 and 2020-25 Dietary Guidelines for Americans food pattern models. *Meats are defined by the Dietary Guidelines for Americans as “(also known as “red meat”)—All forms of beef, pork, lamb, veal, goat, and non-bird game (e.g., venison, bison, elk) and poultry is defined as “All forms of chicken, turkey, duck, geese, guineas, and game birds (e.g., quail, pheasant).”

## Mean intakes (lean oz-eq/week) of red meat, by four different methods, for each age and sex subgroup

| Sex | Age (years) | n | Method 1 (Unprocessed red meat) | Method 2  (Total red meat) | Method 3  (Red and processed meat 1) | Method 4  (Red and processed meat 2) |
| --- | --- | --- | --- | --- | --- | --- |
| **Males** | 2-4 | 459 | 4.3 ± 0.35* | 8 ± 0.5 | 9.8 ± 0.52 | 12 ± 0.55* |
|  | 5-8 | 594 | 6 ± 0.49** | 11.6 ± 0.43 | 13.2 ± 0.53 | 15.2 ± 0.53* |
|  | 9-13 | 740 | 8.1 ± 0.61* | 13.1 ± 0.67 | 14.5 ± 0.68 | 16.5 ± 0.67* |
|  | 14-18 | 715 | 9.8 ± 0.71* | 15.3 ± 0.75 | 16.8 ± 0.85 | 19 ± 0.85* |
|  | 19-30 | 919 | 13.1 ± 0.62** | 19.2 ± 0.78 | 21.3 ± 0.8 | 23.3 ± 0.95* |
|  | 31-50 | 1469 | 15 ± 0.73** | 21.6 ± 0.99 | 24.4 ± 1.08 | 25.6 ± 1.06* |
|  | 51-70 | 1700 | 16.5 ± 0.99* | 23.1 ± 1.13 | 25 ± 1.16 | 25.7 ± 1.19* |
|  | 71+ | 762 | 10.9 ± 0.52** | 18.5 ± 0.7 | 19.9 ± 0.73 | 20.2 ± 0.76 |
|  | 2-18 | 5037 | 7.5 ± 0.4* | 12.5 ± 0.44 | 14.1 ± 0.53 | 16.2 ± 0.5* |
|  | 19+ | 4850 | 14.6 ± 0.49** | 21.2 ± 0.61 | 23.4 ± 0.65 | 24.5 ± 0.7* |
| **Females** | 2-4 | 468 | 3.1 ± 0.27 | 5.5 ± 0.35 | 7 ± 0.36 | 8.9 ± 0.42* |
|  | 5-8 | 586 | 4.4 ± 0.28* | 8.3 ± 0.33 | 9.6 ± 0.41 | 11.3 ± 0.43* |
|  | 9-13 | 770 | 6 ± 0.48* | 9.5 ± 0.59 | 10.7 ± 0.59 | 12.5 ± 0.6* |
|  | 14-18 | 705 | 7.3 ± 0.52* | 11.1 ± 0.62 | 12.4 ± 0.72 | 14.3 ± 0.75* |
|  | 19-30 | 1010 | 8.1 ± 0.55* | 11 ± 0.64 | 12.5 ± 0.63 | 14 ± 0.64* |
|  | 31-50 | 1647 | 8.1 ± 0.52* | 11.8 ± 0.48 | 13.6 ± 0.51 | 14.5 ± 0.49* |
|  | 51-70 | 1745 | 8.9 ± 0.49* | 12.8 ± 0.51 | 14.2 ± 0.61 | 14.8 ± 0.59 |
|  | 71+ | 750 | 7.5 ± 0.43* | 11.5 ± 0.59 | 12.4 ± 0.68 | 12.8 ± 0.62 |
|  | 2-18 | 2529 | 5.5 ± 0.28* | 9 ± 0.38 | 10.3 ± 0.45 | 12.2 ± 0.43* |
|  | 19+ | 5152 | 8.3 ± 0.34* | 11.9 ± 0.33 | 13.4 ± 0.36 | 14.2 ± 0.33 |
| **All** | 2+ | 15039 | 10.2 ± 0.3* | 15.1 ± 0.32 | 16.9 ± 0.35 | 18.1 ± 0.35* |

Mean ± SEM. *Difference from Method 2 (effect size) is ≥ 3.0 oz-eq per week; **difference from Method 2 is ≥ 6.0 oz-eq servings per week; ***difference from Method 2 is ≥ 9.0 oz-eq per week. Method 1: Unprocessed red meat, includes beef, veal, pork, lamb, and game meat; excludes organ meat and processed meat. Method 2: Total red meat, includes unprocessed red meat and processed red meat. Method 3: Red and processed meat, includes unprocessed red meat, processed red meat, and processed poultry. Method 4: Red and processed meat, additionally including chicken patties, nuggets, and tenders. See Figure 1 ontology of red meat methods.

## Mean intakes (lean oz-eq/week) of poultry, by three different methods, for each age and sex subgroup

| Sex | Age (years) | n | Method A  (Unprocessed poultry 1) | Method B (Unprocessed poultry 2) | Method C  (Total poultry) |  |
| --- | --- | --- | --- | --- | --- | --- |
| **Males** | 2-4 | 459 | 4.9 ± 0.4* | 7.1 ± 0.39 | 8.7 ± 0.46 |  |
|  | 5-8 | 594 | 5.4 ± 0.44* | 7.4 ± 0.46 | 9.1 ± 0.53 |  |
|  | 9-13 | 740 | 7.3 ± 0.48* | 9.3 ± 0.54 | 10.8 ± 0.54 |  |
|  | 14-18 | 715 | 9.3 ± 0.77* | 11.4 ± 0.79 | 13.1 ± 0.81 |  |
|  | 19-30 | 919 | 13.9 ± 0.82* | 15.9 ± 0.88 | 17.9 ± 1.01 |  |
|  | 31-50 | 1469 | 12.9 ± 0.76* | 14.1 ± 0.81 | 16.6 ± 0.86 |  |
|  | 51-70 | 1700 | 10 ± 0.59 | 10.8 ± 0.61 | 12.5 ± 0.72 |  |
|  | 71+ | 762 | 7.5 ± 0.6 | 8 ± 0.65 | 9 ± 0.68 |  |
|  | 2-18 | 5037 | 7 ± 0.42* | 9.1 ± 0.45 | 10.7 ± 0.45 |  |
|  | 19+ | 4850 | 11.6 ± 0.46* | 12.7 ± 0.47 | 14.7 ± 0.5 |  |
| **Females** | 2-4 | 468 | 4.6 ± 0.34* | 6.5 ± 0.48 | 7.9 ± 0.57 |  |
|  | 5-8 | 586 | 5.2 ± 0.38* | 7 ± 0.45 | 8.4 ± 0.5 |  |
|  | 9-13 | 770 | 6.9 ± 0.49* | 8.7 ± 0.66 | 10 ± 0.7 |  |
|  | 14-18 | 705 | 8.9 ± 0.82* | 10.7 ± 0.94 | 12 ± 0.98 |  |
|  | 19-30 | 1010 | 9.5 ± 0.56 | 10.9 ± 0.6 | 12.4 ± 0.72 |  |
|  | 31-50 | 1647 | 9.5 ± 0.48 | 10.3 ± 0.51 | 12.2 ± 0.54 |  |
|  | 51-70 | 1745 | 7.8 ± 0.56 | 8.3 ± 0.55 | 9.7 ± 0.59 |  |
|  | 71+ | 750 | 6.9 ± 0.46 | 7.3 ± 0.5 | 8.2 ± 0.52 |  |
|  | 2-18 | 2529 | 6.8 ± 0.44* | 8.6 ± 0.59 | 9.9 ± 0.62 |  |
|  | 19+ | 5152 | 8.6 ± 0.38 | 9.3 ± 0.38 | 10.9 ± 0.42 |  |
| **All** | 2+ | 15039 | 9.3 ± 0.32 | 10.5 ± 0.35 | 12.2 ± 0.36 |  |

Mean ± SEM. *Difference from Method C (effect size) is ≥ 3.0 oz-eq per week; **difference from Method C is ≥ 6.0 oz-eq servings per week; ***difference from Method C is ≥ 9.0 oz-eq per week. Method A: Unprocessed poultry not including chicken patties, nuggets, and tenders. Method B: Unprocessed poultry, includes chicken, turkey, Cornish hens, duck, goose, quail, and pheasant (game birds); excludes organ meat and cured meat and includes chicken patties, nuggets, and tenders. Method C (reference): Total poultry, includes unprocessed poultry, processed poultry, and chicken patties, nuggets, and tenders. See Figure 1 for ontology of poultry methods.

## Intakes (lean oz-eq/week) at the 50^th^ percentile of red meat, by four different methods, for each age and sex subgroup

| Sex | Age (years) | n | Method 1 (Unprocessed red meat) | Method 2  (Total red meat) | Method 3  (Red and processed meat 1) | Method 4  (Red and processed meat 2) | |
| --- | --- | --- | --- | --- | --- | --- | --- |
| **Males** | 2-4 | 459 | 3.9 ± 0.33* | 7.6 ± 0.44 | 9.4 ± 0.48 | 11.6 ± 0.53* |  |
|  | 5-8 | 594 | 5.5 ± 0.51* | 11.2 ± 0.45 | 12.8 ± 0.54 | 14.8 ± 0.51* |  |
|  | 9-13 | 740 | 7.6 ± 0.65* | 12.7 ± 0.7 | 14.1 ± 0.73 | 16.1 ± 0.69* |  |
|  | 14-18 | 715 | 9.2 ± 0.74* | 14.9 ± 0.75 | 16.4 ± 0.85 | 18.7 ± 0.83* |  |
|  | 19-30 | 919 | 12.5 ± 0.69* | 18.4 ± 0.8 | 20.6 ± 0.87 | 22.6 ± 0.98* |  |
|  | 31-50 | 1469 | 14.4 ± 0.74** | 20.8 ± 0.98 | 23.8 ± 1.08 | 24.9 ± 1.04* |  |
|  | 51-70 | 1700 | 15.9 ± 1.03** | 22.3 ± 1.19 | 24.3 ± 1.16 | 25 ± 1.22 |  |
|  | 71+ | 762 | 10.3 ± 0.57** | 17.7 ± 0.8 | 19.2 ± 0.8 | 19.5 ± 0.79 |  |
|  | 2-18 | 5037 | 6.7 ± 0.43* | 12 ± 0.44 | 13.5 ± 0.53 | 15.6 ± 0.47* |  |
|  | 19+ | 4850 | 13.9 ± 0.51** | 20.3 ± 0.67 | 22.6 ± 0.69 | 23.7 ± 0.72* |  |
| **Females** | 2-4 | 468 | 2.7 ± 0.24 | 5.2 ± 0.31 | 6.6 ± 0.35 | 8.5 ± 0.41* |  |
|  | 5-8 | 586 | 4 ± 0.3* | 7.9 ± 0.4 | 9.2 ± 0.48 | 10.9 ± 0.47* |  |
|  | 9-13 | 770 | 5.5 ± 0.5* | 9.1 ± 0.65 | 10.3 ± 0.68 | 12.1 ± 0.66* |  |
|  | 14-18 | 705 | 6.7 ± 0.55* | 10.7 ± 0.66 | 11.9 ± 0.77 | 14 ± 0.77* |  |
|  | 19-30 | 1010 | 7.5 ± 0.56 | 10.5 ± 0.66 | 12 ± 0.65 | 13.6 ± 0.69* |  |
|  | 31-50 | 1647 | 7.5 ± 0.56* | 11.4 ± 0.52 | 13.1 ± 0.56 | 14.1 ± 0.54 |  |
|  | 51-70 | 1745 | 8.3 ± 0.52* | 12.3 ± 0.54 | 13.7 ± 0.66 | 14.4 ± 0.66 |  |
|  | 71+ | 750 | 6.9 ± 0.47* | 11 ± 0.66 | 11.9 ± 0.75 | 12.3 ± 0.71 |  |
|  | 2-18 | 2529 | 4.9 ± 0.29* | 8.5 ± 0.42 | 9.7 ± 0.49 | 11.6 ± 0.45* |  |
|  | 19+ | 5152 | 7.7 ± 0.38* | 11.4 ± 0.38 | 12.9 ± 0.43 | 13.8 ± 0.41 |  |
| **All** | 2+ | 15039 | 8.9 ± 0.29* | 13.6 ± 0.32 | 15.4 ± 0.35 | 16.7 ± 0.34* |  |

Mean ± SEM. *Difference from Method 2 (effect size) is ≥ 3.0 oz-eq per week; **difference from Method 2 is ≥ 6.0 oz-eq servings per week; ***difference from Method 2 is ≥ 9.0 oz-eq per week. Method 1: Unprocessed red meat, includes beef, veal, pork, lamb, and game meat; excludes organ meat and processed meat. Method 2: Total red meat, includes unprocessed red meat and processed red meat. Method 3: Red and processed meat, includes unprocessed red meat, processed red meat, and processed poultry. Method 4: Red and processed meat, additionally including chicken patties, nuggets, and tenders. See Figure 1 ontology of red meat methods.

## Intakes (lean oz-eq/week) at the 95^th^ percentile of red meat, by four different methods, for each age and sex subgroup

| Sex | Age (years) | n | Method 1  (Unprocessed red meat) | Method 2  (Total red meat) | Method 3  (Red and processed meat 1) | Method 4  (Red and processed meat 2) |
| --- | --- | --- | --- | --- | --- | --- |
| **Males** | 2-4 | 459 | 8.6 ± 0.91* | 14.1 ± 1.31 | 17.3 ± 1.34* | 20.3 ± 1.34** |
|  | 5-8 | 594 | 11.5 ± 0.96** | 19.4 ± 1.2 | 22.3 ± 1.39 | 25 ± 1.44* |
|  | 9-13 | 740 | 15.2 ± 1.15** | 21.5 ± 1.41 | 24.1 ± 1.45 | 26.7 ± 1.48* |
|  | 14-18 | 715 | 18 ± 1.41** | 25.2 ± 1.66 | 28 ± 1.85 | 30.9 ± 1.85* |
|  | 19-30 | 919 | 23.3 ± 1.48*** | 33.9 ± 1.7 | 35.8 ± 1.26 | 38.3 ± 1.46* |
|  | 31-50 | 1469 | 26 ± 2*** | 37.5 ± 2.03 | 40.1 ± 1.98 | 41.6 ± 1.94* |
|  | 51-70 | 1700 | 28.1 ± 2.37*** | 39 ± 2.19 | 40.4 ± 2.1 | 41.4 ± 1.96 |
|  | 71+ | 762 | 19.7 ± 1.41*** | 32 ± 1.32 | 32.9 ± 1.2 | 33.4 ± 1.22 |
|  | 2-18 | 5037 | 15.3 ± 0.99** | 22.2 ± 1.22 | 24.8 ± 1.38 | 27.6 ± 1.39* |
|  | 19+ | 4850 | 25.9 ± 1.82*** | 36.9 ± 1.64 | 38.9 ± 1.52 | 40.3 ± 1.49* |
| **Females** | 2-4 | 468 | 6.3 ± 0.76* | 10.4 ± 0.97 | 13.1 ± 1.01 | 16 ± 1.09* |
|  | 5-8 | 586 | 8.7 ± 0.71* | 14.6 ± 0.85 | 17 ± 0.99 | 19.6 ± 1.08* |
|  | 9-13 | 770 | 11.6 ± 1.1* | 16.5 ± 1.18 | 18.8 ± 1.21 | 21.5 ± 1.23* |
|  | 14-18 | 705 | 13.9 ± 1.19* | 19.4 ± 1.22 | 21.9 ± 1.39 | 24.8 ± 1.44* |
|  | 19-30 | 1010 | 15.4 ± 1.17* | 20.5 ± 1.24 | 22.8 ± 1.25 | 24.5 ± 1.28* |
|  | 31-50 | 1647 | 15.2 ± 0.99** | 21.6 ± 1.06 | 24.1 ± 1.1 | 25.1 ± 1.27* |
|  | 51-70 | 1745 | 16.3 ± 0.99** | 23 ± 1.18 | 25.1 ± 1.31 | 25.5 ± 1.34 |
|  | 71+ | 750 | 14 ± 0.87** | 20.8 ± 1.09 | 22.1 ± 1.22 | 22.4 ± 1.22 |
|  | 2-18 | 2529 | 11.7 ± 0.87* | 16.9 ± 0.95 | 19.3 ± 1.08 | 22 ± 1.13* |
|  | 19+ | 5152 | 15.5 ± 0.83** | 21.9 ± 0.97 | 24 ± 1.04 | 24.8 ± 1.12 |
| **All** | 2+ | 15039 | 21.9 ± 1.14*** | 31.1 ± 1.04 | 33.3 ± 1.02 | 34.6 ± 1.05* |

Mean ± SEM. *Difference from Method 2 (effect size) is ≥ 3.0 oz-eq per week; **difference from Method 2 is ≥ 6.0 oz-eq servings per week; ***difference from Method 2 is ≥ 9.0 oz-eq per week. Method 1: Unprocessed red meat, includes beef, veal, pork, lamb, and game meat; excludes organ meat and processed meat. Method 2: Total red meat, includes unprocessed red meat and processed red meat. Method 3: Red and processed meat, includes unprocessed red meat, processed red meat, and processed poultry. Method 4: Red and processed meat, additionally including chicken patties, nuggets, and tenders. See Figure 1 ontology of red meat methods.

## Intakes (lean oz-eq/week) at the 50^th^ percentile of poultry, by three different methods, for each age and sex subgroup

| Sex | Age (years) | n | Method A  (Unprocessed poultry 1) | Method B  (Unprocessed poultry 2) | Method C  (Total poultry) |  |
| --- | --- | --- | --- | --- | --- | --- |
| **Males** | 2-4 | 459 | 4.4 ± 0.38* | 6.7 ± 0.4 | 8.3 ± 0.46 |  |
|  | 5-8 | 594 | 4.8 ± 0.47* | 6.8 ± 0.49 | 8.6 ± 0.51 |  |
|  | 9-13 | 740 | 6.6 ± 0.49* | 8.6 ± 0.57 | 10.1 ± 0.58 |  |
|  | 14-18 | 715 | 8.5 ± 0.84* | 10.6 ± 0.87 | 12.3 ± 0.89 |  |
|  | 19-30 | 919 | 12.6 ± 0.85* | 14.7 ± 0.9 | 16.9 ± 1.05 |  |
|  | 31-50 | 1469 | 11.7 ± 0.78* | 12.9 ± 0.79 | 15.5 ± 0.83 |  |
|  | 51-70 | 1700 | 8.9 ± 0.62 | 9.5 ± 0.64 | 11.3 ± 0.79 |  |
|  | 71+ | 762 | 6.6 ± 0.55 | 7 ± 0.6 | 8 ± 0.63 |  |
|  | 2-18 | 5037 | 6.1 ± 0.43* | 8.2 ± 0.46 | 9.9 ± 0.46 |  |
|  | 19+ | 4850 | 10.3 ± 0.5* | 11.4 ± 0.49 | 13.4 ± 0.53 |  |
| **Females** | 2-4 | 468 | 4.2 ± 0.34* | 6.2 ± 0.49 | 7.6 ± 0.58 |  |
|  | 5-8 | 586 | 4.8 ± 0.44* | 6.5 ± 0.5 | 8 ± 0.51 |  |
|  | 9-13 | 770 | 6.3 ± 0.52* | 8.2 ± 0.7 | 9.4 ± 0.73 |  |
|  | 14-18 | 705 | 8.2 ± 0.9* | 10.1 ± 1.02 | 11.4 ± 1.05 |  |
|  | 19-30 | 1010 | 9 ± 0.63* | 10.3 ± 0.67 | 12 ± 0.78 |  |
|  | 31-50 | 1647 | 8.9 ± 0.55 | 9.8 ± 0.59 | 11.8 ± 0.61 |  |
|  | 51-70 | 1745 | 7.2 ± 0.63 | 7.6 ± 0.63 | 9.1 ± 0.62 |  |
|  | 71+ | 750 | 6.3 ± 0.52 | 6.7 ± 0.55 | 7.7 ± 0.59 |  |
|  | 2-18 | 2529 | 6 ± 0.46* | 7.8 ± 0.59 | 9.2 ± 0.62 |  |
|  | 19+ | 5152 | 8 ± 0.46 | 8.7 ± 0.46 | 10.4 ± 0.47 |  |
| **All** | 2+ | 15039 | 8.2 ± 0.35 | 9.3 ± 0.38 | 11 ± 0.37 |  |

Mean ± SEM. *Difference from Method C (effect size) is ≥ 3.0 oz-eq per week; **difference from Method C is ≥ 6.0 oz-eq servings per week; ***difference from Method C is ≥ 9.0 oz-eq per week. Method A: Unprocessed poultry not including chicken patties, nuggets, and tenders. Method B: Unprocessed poultry, includes chicken, turkey, Cornish hens, duck, goose, quail, and pheasant (game birds); excludes organ meat and cured meat and includes chicken patties, nuggets, and tenders. Method C (reference): Total poultry, includes unprocessed poultry, processed poultry, and chicken patties, nuggets, and tenders. See Figure 1 for ontology of poultry methods.

## Intakes (lean oz-eq/week) at the 95^th^ percentile of poultry, by three different methods, for each age and sex subgroup

| Sex | Age (years) | n | Method A  (Unprocessed poultry 1) | Method B  (Unprocessed poultry 2) | Method C  (Total poultry) |
| --- | --- | --- | --- | --- | --- |
| **Males** | 2-4 | 459 | 9.9 ± 1.09* | 13.1 ± 1.03 | 15.2 ± 1.23 |
|  | 5-8 | 594 | 11.1 ± 0.98* | 14.5 ± 0.97 | 16.5 ± 1.33 |
|  | 9-13 | 740 | 14.7 ± 1.32* | 17.8 ± 1.29 | 19.7 ± 1.38 |
|  | 14-18 | 715 | 18.3 ± 1.51* | 21.7 ± 1.29 | 23.8 ± 1.45 |
|  | 19-30 | 919 | 27.6 ± 1.98** | 30.6 ± 2.09 | 33.6 ± 2.31 |
|  | 31-50 | 1469 | 25.8 ± 1.86* | 27.7 ± 2.05* | 31.3 ± 2.17 |
|  | 51-70 | 1700 | 21.3 ± 1.57* | 22.7 ± 1.66 | 25.3 ± 1.55 |
|  | 71+ | 762 | 16.5 ± 1.62 | 17.4 ± 1.66 | 19.2 ± 1.66 |
|  | 2-18 | 5037 | 15.2 ± 1.12* | 18.3 ± 0.99 | 20.3 ± 1.14 |
|  | 19+ | 4850 | 24.5 ± 1.5* | 26.6 ± 1.57* | 29.7 ± 1.65 |
| **Females** | 2-4 | 468 | 9 ± 0.93* | 11.8 ± 1.03 | 13.8 ± 1.24 |
|  | 5-8 | 586 | 10.5 ± 0.78* | 13.4 ± 0.93 | 15.1 ± 1.17 |
|  | 9-13 | 770 | 13.7 ± 1.23* | 16.4 ± 1.33 | 18.1 ± 1.43 |
|  | 14-18 | 705 | 17 ± 1.37* | 20 ± 1.44 | 21.8 ± 1.57 |
|  | 19-30 | 1010 | 17.3 ± 1.1* | 19.4 ± 1.24 | 21.3 ± 1.36 |
|  | 31-50 | 1647 | 17.2 ± 0.96* | 18.8 ± 1 | 20.9 ± 1.15 |
|  | 51-70 | 1745 | 14.8 ± 0.94* | 16 ± 0.91 | 17.9 ± 1.2 |
|  | 71+ | 750 | 13.2 ± 0.86 | 14 ± 0.9 | 15.4 ± 0.86 |
|  | 2-18 | 2529 | 14.3 ± 1.03* | 17 ± 1.15 | 18.7 ± 1.25 |
|  | 19+ | 5152 | 16.2 ± 0.81* | 17.8 ± 0.85 | 19.8 ± 1.01 |
| **All** | 2+ | 15039 | 19.9 ± 0.72* | 21.9 ± 0.77 | 24.6 ± 0.86 |

Mean ± SEM. *Difference from Method C (effect size) is ≥ 3.0 oz-eq per week; **difference from Method C is ≥ 6.0 oz-eq servings per week; ***difference from Method C is ≥ 9.0 oz-eq per week. Method A: Unprocessed poultry not including chicken patties, nuggets, and tenders. Method B: Unprocessed poultry, includes chicken, turkey, Cornish hens, duck, goose, quail, and pheasant (game birds); excludes organ meat and cured meat and includes chicken patties, nuggets, and tenders. Method C (reference): Total poultry, includes unprocessed poultry, processed poultry, and chicken patties, nuggets, and tenders. See Figure 1 for ontology of poultry methods.

## Percent of the population below age- and sex-specific ‘meat’, i.e. red meat, allotment ranges from the 2020-2025 Dietary Guidelines for Americans recommended eating patterns, by four different red meat food group methods

| Sex | Age (years) | n | Method 1  (Unprocessed red meat) | Method 2  (Total red meat) | Method 3  (Red and processed meat 1) | Method 4  (Red and processed meat 2) |  |
| --- | --- | --- | --- | --- | --- | --- | --- |
|  |  |  |  |  |  |  |  |
| **Males** | 2-4 | 459 | 68 ± 6*** | 19 ± 3.4 | 11 ± 2.5 | 5 ± 1.5* |  |
|  | 5-8 | 594 | 69 ± 6.1*** | 14 ± 3.4 | 10 ± 2.5 | 5 ± 1.4 |  |
|  | 9-13 | 740 | 80 ± 5.1*** | 36 ± 5.9 | 27 ± 5.1 | 17 ± 3.5* |  |
|  | 14-18 | 715 | 79 ± 5.2*** | 36 ± 5.3 | 29 ± 4.9 | 19 ± 3.4* |  |
|  | 19-30 | 919 | 66 ± 4.1*** | 33 ± 3.9 | 23 ± 3.9* | 16 ± 3.5* |  |
|  | 31-50 | 1469 | 47 ± 4.7** | 20 ± 3.4 | 11 ± 2.5 | 9 ± 2.1* |  |
|  | 51-70 | 1700 | 32 ± 5.4** | 11 ± 3 | 7 ± 2.1 | 6 ± 1.9 |  |
|  | 71+ | 762 | 70 ± 4.1*** | 24 ± 4.5 | 17 ± 3.9 | 16 ± 3.6 |  |
|  | 2-18 | 2508 | 75 ± 5*** | 28 ± 3.9 | 21 ± 3.6 | 13 ± 2.3* |  |
|  | 19+ | 4850 | 49 ± 3.1** | 20 ± 2.7 | 13 ± 2.3 | 10 ± 2* |  |
| **Females** | 2-4 | 468 | 87 ± 4.8*** | 46 ± 4.8 | 30 ± 3.9* | 16 ± 2.8*** |  |
|  | 5-8 | 586 | 87 ± 3.4*** | 39 ± 4.9 | 28 ± 4.6* | 18 ± 3.2** |  |
|  | 9-13 | 770 | 85 ± 4.5*** | 50 ± 6.5 | 39 ± 5.9* | 26 ± 4.6** |  |
|  | 14-18 | 705 | 86 ± 4.2*** | 53 ± 5.6 | 43 ± 5.8* | 31 ± 4.9** |  |
|  | 19-30 | 1010 | 80 ± 4.6** | 54 ± 4.9 | 43 ± 4.4* | 33 ± 4.5** |  |
|  | 31-50 | 1647 | 80 ± 4.1*** | 47 ± 3.7 | 36 ± 3.9* | 29 ± 3.6* |  |
|  | 51-70 | 1745 | 73 ± 4.2*** | 41 ± 3.6 | 32 ± 3.9 | 29 ± 3.7* |  |
|  | 71+ | 750 | 85 ± 3.2*** | 50 ± 5 | 43 ± 5.7 | 40 ± 5.5* |  |
|  | 2-18 | 2529 | 86 ± 3.2*** | 48 ± 4.2 | 36 ± 4.4* | 24 ± 3.4** |  |
|  | 19+ | 5152 | 78 ± 2.8*** | 47 ± 2.7 | 37 ± 2.9* | 31 ± 3* |  |
| **All** | 2+ | 15039 | 68 ± 2.4*** | 35 ± 2 | 26 ± 1.8 | 21 ± 1.6* |  |

Mean ± SEM. *Difference from Method 2 (effect size) is ≥10%; **difference from Method 2 is ≥20%; ***difference from Method 2 is ≥30%. Method 1: Unprocessed red meat, includes beef, veal, pork, lamb, and game meat; excludes organ meat and processed meat. Method 2: Total red meat, includes unprocessed red meat and processed red meat. Method 3: Red and processed meat, includes unprocessed red meat, processed red meat, and processed poultry. Method 4: Red and processed meat, additionally including chicken patties, nuggets, and tenders. See Figure 1 for ontology of red meat methods.

## Percent of the population within age- and sex-specific ‘meat’, i.e. red meat, allotment ranges from the 2020-2025 Dietary Guidelines for Americans recommended eating patterns, by four different methods

| Sex | Age (years) | n | Method 1  (Unprocessed red meat) | Method 2  (Total red meat) | Method 3  (Red and processed meat 1) | Method 4  (Red and processed meat 2) |  |
| --- | --- | --- | --- | --- | --- | --- | --- |
|  |  |  |  |  |  |  |  |
| **Males** | 2-4 | 459 | 31 ± 5.3 | 63 ± 4.7 | 54 ± 4 | 39 ± 3.6 |  |
|  | 5-8 | 594 | 29 ± 5.3 | 53 ± 3.8 | 42 ± 3.2 | 31 ± 3 |  |
|  | 9-13 | 740 | 15 ± 3.5 | 32 ± 3.4 | 29 ± 2.5 | 25 ± 2.1 |  |
|  | 14-18 | 715 | 12 ± 2.2 | 22 ± 2.2 | 19 ± 1.6 | 15 ± 1.4 |  |
|  | 19-30 | 919 | 6 ± 0.9 | 5 ± 0.3 | 5 ± 0.4 | 4 ± 0.4 |  |
|  | 31-50 | 1469 | 13 ± 1.5 | 8 ± 0.8 | 6 ± 0.8 | 5 ± 0.7 |  |
|  | 51-70 | 1700 | 18 ± 2.5 | 10 ± 1.8 | 8 ± 1.6 | 7 ± 1.6 |  |
|  | 71+ | 762 | 11 ± 1.5 | 10 ± 0.6 | 9 ± 0.7 | 9 ± 0.8 |  |
|  | 2-18 | 2508 | 20 ± 3.8* | 39 ± 3.3 | 33 ± 2.2 | 26 ± 1.8* |  |
|  | 19+ | 4850 | 13 ± 1.5 | 8 ± 0.7 | 7 ± 0.7 | 6 ± 0.7 |  |
| **Females** | 2-4 | 468 | 12 ± 4.2*** | 44 ± 3.7 | 45 ± 3.8 | 39 ± 3 |  |
|  | 5-8 | 586 | 11 ± 2.7** | 40 ± 4.4 | 38 ± 3.2 | 33 ± 2.2 |  |
|  | 9-13 | 770 | 13 ± 3.6** | 37 ± 4.8 | 39 ± 4.3 | 38 ± 2.8 |  |
|  | 14-18 | 705 | 11 ± 2.6* | 28 ± 3.5 | 28 ± 2.9 | 26 ± 1.9 |  |
|  | 19-30 | 1010 | 15 ± 2.7* | 25 ± 2 | 26 ± 2 | 26 ± 1.8 |  |
|  | 31-50 | 1647 | 13 ± 2.1 | 21 ± 1.6 | 21 ± 1.3 | 20 ± 1.1 |  |
|  | 51-70 | 1745 | 16 ± 2.1 | 20 ± 1.2 | 20 ± 1.2 | 19 ± 1.3 |  |
|  | 71+ | 750 | 8 ± 1.3 | 15 ± 1.4 | 15 ± 1.3 | 15 ± 1.3 |  |
|  | 2-18 | 2529 | 12 ± 2.4** | 36 ± 3.8 | 36 ± 3.5 | 33 ± 2.2 |  |
|  | 19+ | 5152 | 13 ± 1.4 | 21 ± 1.4 | 21 ± 1.3 | 20 ± 1.2 |  |
| **All** | 2+ | 15039 | 14 ± 0.9 | 20 ± 1.1 | 19 ± 0.9 | 17 ± 0.7 |  |

Mean ± SEM. *Difference from Method 2 (effect size) is ≥10%; **difference from Method 2 is ≥20%; ***difference from Method 2 is ≥30%. Method 1: Unprocessed red meat, includes beef, veal, pork, lamb, and game meat; excludes organ meat and processed meat. Method 2: Total red meat, includes unprocessed red meat and processed red meat. Method 3: Red and processed meat, includes unprocessed red meat, processed red meat, and processed poultry. Method 4: Red and processed meat, additionally including chicken patties, nuggets, and tenders. See Figure 1 for ontology of red meat methods.

## Percent of the population above age- and sex-specific ‘meat’, i.e. red meat, allotment ranges from the 2020-2025 Dietary Guidelines for Americans recommended eating patterns, by four different methods

| Sex | Age (years) | n | Method 1  (Unprocessed red meat) | Method 2  (Total red meat) | Method 3  (Red and processed meat 1) | Method 4  (Red and processed meat 2) |  |
| --- | --- | --- | --- | --- | --- | --- | --- |
| **Males** | 2-4 | 459 | 1 ± 0.9* | 18 ± 5.1 | 35 ± 4.7* | 56 ± 4.6*** |  |
|  | 5-8 | 594 | 2 ± 1.4*** | 34 ± 3.9 | 48 ± 4.4* | 64 ± 3.9*** |  |
|  | 9-13 | 740 | 5 ± 2.1** | 32 ± 5.1 | 43 ± 5.1* | 58 ± 4.7** |  |
|  | 14-18 | 715 | 9 ± 3.3*** | 42 ± 5.2 | 52 ± 5.4* | 65 ± 4.5** |  |
|  | 19-30 | 919 | 28 ± 3.9*** | 62 ± 4 | 72 ± 4.2* | 80 ± 3.9* |  |
|  | 31-50 | 1469 | 40 ± 4.8*** | 72 ± 4.1 | 83 ± 3.2* | 86 ± 2.7* |  |
|  | 51-70 | 1700 | 49 ± 6.4*** | 79 ± 4.6 | 86 ± 3.6 | 88 ± 3.4 |  |
|  | 71+ | 762 | 19 ± 3.5*** | 65 ± 4.8 | 73 ± 4.4 | 75 ± 4.2 |  |
|  | 2-18 | 2508 | 2 ± 2.8** | 33 ± 3.4 | 46 ± 3.8* | 61 ± 3.4** |  |
|  | 19+ | 4850 | 38 ± 3.4*** | 71 ± 3.2 | 80 ± 2.9 | 84 ± 2.6* |  |
| **Females** | 2-4 | 468 | 1 ± 0.6* | 10 ± 3.8 | 25 ± 3.9* | 45 ± 4.3*** |  |
|  | 5-8 | 586 | 1 ± 0.8* | 20 ± 3.1 | 34 ± 4* | 50 ± 4.1*** |  |
|  | 9-13 | 770 | 2 ± 1.1* | 13 ± 3.9 | 22 ± 4.1 | 36 ± 4.5** |  |
|  | 14-18 | 705 | 3 ± 1.7* | 19 ± 4 | 29 ± 4.7* | 43 ± 5.1** |  |
|  | 19-30 | 1010 | 6 ± 2.2* | 21 ± 3.9 | 31 ± 4* | 41 ± 4.3* |  |
|  | 31-50 | 1647 | 8 ± 2.4** | 32 ± 3.1 | 44 ± 3.5* | 51 ± 3.5* |  |
|  | 51-70 | 1745 | 11 ± 2.7** | 39 ± 3.6 | 48 ± 4.2 | 52 ± 4.3* |  |
|  | 71+ | 750 | 7 ± 2.2** | 35 ± 4.5 | 42 ± 5.4 | 45 ± 5.2* |  |
|  | 2-18 | 2529 | 2 ± 1* | 16 ± 3 | 27 ± 3.3* | 43 ± 3.4** |  |
|  | 19+ | 5152 | 8 ± 1.9** | 33 ± 2.2 | 42 ± 2.5* | 48 ± 2.7* |  |
| **All** | 2+ | 15039 | 18 ± 1.9** | 45 ± 1.8 | 55 ± 1.8* | 62 ± 1.8* |  |

Mean ± SEM. *Difference from Method 2 (effect size) is ≥10%; **difference from Method 2 is ≥20%; ***difference from Method 2 is ≥30%. Method 1: Unprocessed red meat, includes beef, veal, pork, lamb, and game meat; excludes organ meat and processed meat. Method 2: Total red meat, includes unprocessed red meat and processed red meat. Method 3: Red and processed meat, includes unprocessed red meat, processed red meat, and processed poultry. Method 4: Red and processed meat, additionally including chicken patties, nuggets, and tenders. See Figure 1 for ontology of red meat methods.

## Percent of the population below age- and sex-specific poultry allotment ranges from the 2020-2025 Dietary Guidelines for Americans recommended eating patterns, by three different methods

| Sex | Age (years) | n | Method A  (Unprocessed poultry 1) | Method B  (Unprocessed poultry 2) | Method C  (Total poultry) |
| --- | --- | --- | --- | --- | --- |
| **Males** | 2-4 | 459 | 42 ± 6.3*** | 17 ± 4* | 7 ± 2.7 |
|  | 5-8 | 594 | 66 ± 5.5*** | 41 ± 5.8* | 23 ± 5.3 |
|  | 9-13 | 740 | 72 ± 4.2*** | 53 ± 5* | 40 ± 5 |
|  | 14-18 | 715 | 62 ± 6.5** | 46 ± 6.3* | 34 ± 6.4 |
|  | 19-30 | 919 | 46 ± 4.8** | 35 ± 4.8 | 26 ± 4.3 |
|  | 31-50 | 1469 | 46 ± 4.8* | 39 ± 4.5* | 27 ± 3.5 |
|  | 51-70 | 1700 | 58 ± 4.3* | 53 ± 4.4* | 42 ± 4.8 |
|  | 71+ | 762 | 76 ± 4.4* | 72 ± 4.7 | 64 ± 4.8 |
|  | 2-18 | 5037 | 62 ± 4.2*** | 42 ± 4.3* | 29 ± 4.4 |
|  | 19+ | 4850 | 53 ± 3* | 46 ± 2.9* | 36 ± 2.7 |
| **Females** | 2-4 | 468 | 46 ± 6.1*** | 21 ± 5.2* | 10 ± 4 |
|  | 5-8 | 586 | 67 ± 5.1*** | 44 ± 6* | 28 ± 5.9 |
|  | 9-13 | 770 | 67 ± 4.9*** | 48 ± 6.7* | 37 ± 6.7 |
|  | 14-18 | 705 | 57 ± 8** | 42 ± 7.8* | 32 ± 7.7 |
|  | 19-30 | 1010 | 51 ± 5.9** | 39 ± 5* | 27 ± 4.7 |
|  | 31-50 | 1647 | 51 ± 5.4** | 43 ± 5.1* | 28 ± 4.1 |
|  | 51-70 | 1745 | 68 ± 5.8* | 63 ± 5.5* | 49 ± 5.3 |
|  | 71+ | 750 | 77 ± 4.7* | 72 ± 4.9* | 62 ± 5.3 |
|  | 2-18 | 2529 | 60 ± 5*** | 40 ± 5.9* | 29 ± 5.8 |
|  | 19+ | 5152 | 60 ± 4.3* | 53 ± 3.9* | 39 ± 3.5 |
| **All** | 2+ | 15039 | 58 ± 2.9** | 48 ± 2.8* | 36 ± 2.6 |

Mean ± SEM. *Difference from Method C (effect size) is ≥10%; **difference from Method C is ≥20%; ***difference from Method C is ≥30%. Method A: Unprocessed poultry not including chicken patties, nuggets, and tenders. Method B: Unprocessed poultry, includes chicken, turkey, Cornish hens, duck, goose, quail, and pheasant (game birds); excludes organ meat and cured meat and includes chicken patties, nuggets, and tenders. Method C (reference): Total poultry, includes unprocessed poultry, processed poultry, and chicken patties, nuggets, and tenders. See Figure 1 for ontology of poultry methods.

## Percent of the population within age- and sex-specific poultry allotment ranges from the 2020-2025 Dietary Guidelines for Americans recommended eating patterns, by three different methods

| Sex | Age (years) | n | Method A  (Unprocessed poultry 1) | Method B  (Unprocessed poultry 2) | Method C  (Total poultry) |  |
| --- | --- | --- | --- | --- | --- | --- |
| **Males** | 2-4 | 459 | 50 ± 5.9 | 57 ± 5 | 51 ± 4.4 |  |
|  | 5-8 | 594 | 26 ± 4.4* | 36 ± 4.3 | 40 ± 3.8 |  |
|  | 9-13 | 740 | 16 ± 2.1 | 21 ± 2.3 | 24 ± 2.6 |  |
|  | 14-18 | 715 | 18 ± 3 | 20 ± 2.2 | 21 ± 1.9 |  |
|  | 19-30 | 919 | 6 ± 0.6 | 6 ± 0.6 | 5 ± 0.5 |  |
|  | 31-50 | 1469 | 12 ± 1.3 | 12 ± 1.2 | 10 ± 0.9 |  |
|  | 51-70 | 1700 | 17 ± 2 | 17 ± 2 | 18 ± 1.3 |  |
|  | 71+ | 762 | 9 ± 1 | 10 ± 1.1 | 11 ± 0.9 |  |
|  | 2-18 | 5037 | 25 ± 3.2 | 30 ± 3.1 | 31 ± 2.7 |  |
|  | 19+ | 4850 | 12 ± 1.2 | 12 ± 1.1 | 12 ± 0.7 |  |
| **Females** | 2-4 | 468 | 45 ± 5.8 | 51 ± 5 | 46 ± 5 |  |
|  | 5-8 | 586 | 23 ± 4.1* | 31 ± 3.7 | 33 ± 3.3 |  |
|  | 9-13 | 770 | 20 ± 3 | 25 ± 2.9 | 27 ± 2.9 |  |
|  | 14-18 | 705 | 20 ± 3.5 | 22 ± 2.3 | 23 ± 2.1 |  |
|  | 19-30 | 1010 | 24 ± 2.3 | 24 ± 2.2 | 23 ± 2.3 |  |
|  | 31-50 | 1647 | 17 ± 1.5 | 17 ± 1.3 | 16 ± 1.4 |  |
|  | 51-70 | 1745 | 14 ± 1.9 | 14 ± 1.8 | 16 ± 1.2 |  |
|  | 71+ | 750 | 7 ± 1 | 7 ± 0.9 | 8 ± 0.9 |  |
|  | 2-18 | 2529 | 25 ± 3.6 | 30 ± 3.1 | 30 ± 2.8 |  |
|  | 19+ | 5152 | 16 ± 1.6 | 16 ± 1.4 | 16 ± 1.2 |  |
| **All** | 2+ | 15039 | 16 ± 1.2 | 18 ± 1 | 18 ± 0.9 |  |

Mean ± SEM. *Difference from Method C (effect size) is ≥10%; **difference from Method C is ≥20%; ***difference from Method C is ≥30%. Method A: Unprocessed poultry not including chicken patties, nuggets, and tenders. Method B: Unprocessed poultry, includes chicken, turkey, Cornish hens, duck, goose, quail, and pheasant (game birds); excludes organ meat and cured meat and includes chicken patties, nuggets, and tenders. Method C (reference): Total poultry, includes unprocessed poultry, processed poultry, and chicken patties, nuggets, and tenders. See Figure 1 for ontology of poultry methods.

## Percent of the population above age- and sex-specific poultry allotment ranges from the 2020-2025 Dietary Guidelines for Americans recommended eating patterns, by three different methods

| Sex | Age (years) | n | Method A  (Unprocessed poultry 1) |  | Method B  (Unprocessed poultry 2) | Method C  (Total poultry) |
| --- | --- | --- | --- | --- | --- | --- |
| **Males** | 2-4 | 459 | 8 ± 3.5*** |  | 26 ± 4.6* | 42 ± 5.2 |
|  | 5-8 | 594 | 8 ± 2.6** |  | 22 ± 3.5* | 37 ± 4.7 |
|  | 9-13 | 740 | 12 ± 3.2** |  | 25 ± 4.3* | 36 ± 4.4 |
|  | 14-18 | 715 | 20 ± 4.5** |  | 35 ± 5.1* | 46 ± 5.9 |
|  | 19-30 | 919 | 48 ± 4.6** |  | 59 ± 4.7 | 69 ± 4.6 |
|  | 31-50 | 1469 | 42 ± 4.6** |  | 49 ± 4.4* | 63 ± 3.9 |
|  | 51-70 | 1700 | 25 ± 3.4* |  | 30 ± 3.7* | 40 ± 4.5 |
|  | 71+ | 762 | 15 ± 3.7 |  | 18 ± 4 | 25 ± 4.4 |
|  | 2-18 | 5037 | 13 ± 2.8** |  | 27 ± 3.1* | 40 ± 3.5 |
|  | 19+ | 4850 | 35 ± 2.7* |  | 42 ± 2.5* | 53 ± 2.7 |
| **Females** | 2-4 | 468 | 9 ± 3.6*** |  | 28 ± 6.2* | 45 ± 7.4 |
|  | 5-8 | 586 | 10 ± 2.5** |  | 25 ± 4.3* | 39 ± 5.3 |
|  | 9-13 | 770 | 13 ± 3.5** |  | 27 ± 5.6 | 36 ± 6.1 |
|  | 14-18 | 705 | 22 ± 5.5** |  | 36 ± 7.1 | 46 ± 7.7 |
|  | 19-30 | 1010 | 25 ± 5** |  | 37 ± 5.4* | 50 ± 6 |
|  | 31-50 | 1647 | 32 ± 4.8** |  | 40 ± 4.8* | 56 ± 4.8 |
|  | 51-70 | 1745 | 19 ± 4.4* |  | 23 ± 4.3* | 35 ± 5.1 |
|  | 71+ | 750 | 17 ± 3.9* |  | 21 ± 4.2 | 30 ± 4.8 |
|  | 2-18 | 2529 | 15 ± 3.1*** |  | 30 ± 5* | 41 ± 5.6 |
|  | 19+ | 5152 | 24 ± 3.6** |  | 31 ± 3.5* | 44 ± 3.8 |
| **All** | 2+ | 15039 | 26 ± 2.2** |  | 34 ± 2.4* | 47 ± 2.6 |

Mean ± SEM. *Difference from Method C (effect size) is ≥10%; **difference from Method C is ≥20%; ***difference from Method C is ≥30%. Method A: Unprocessed poultry not including chicken patties, nuggets, and tenders. Method B: Unprocessed poultry, includes chicken, turkey, Cornish hens, duck, goose, quail, and pheasant (game birds); excludes organ meat and cured meat and includes chicken patties, nuggets, and tenders. Method C (reference): Total poultry, includes unprocessed poultry, processed poultry, and chicken patties, nuggets, and tenders. See Figure 1 for ontology of poultry methods.
